# A novel form of Deleted in breast cancer 1 (DBC1) lacking the N-terminal domain does not bind SIRT1 and is dynamically regulated *in vivo*

**DOI:** 10.1038/s41598-019-50789-7

**Published:** 2019-10-07

**Authors:** Leonardo Santos, Laura Colman, Paola Contreras, Claudia C. Chini, Adriana Carlomagno, Alejandro Leyva, Mariana Bresque, Inés Marmisolle, Celia Quijano, Rosario Durán, Florencia Irigoín, Victoria Prieto-Echagüe, Mikkel H. Vendelbo, José R. Sotelo-Silveira, Eduardo N. Chini, Jose L. Badano, Aldo J. Calliari, Carlos Escande

**Affiliations:** 1 Laboratory of Metabolic Diseases and Aging, INDICyO Program, Institut Pasteur de Montevideo, Montevideo, Uruguay; 2 Signal Transduction and Molecular Nutrition Laboratory, Kogod Aging Center, Department of Anesthesiology and Perioperative Medicine, Mayo Clinic College of Medicine, Rochester, USA; 3 Departamento de Fisiología, Facultad de Medicina, Universidad de la República, Montevideo, Uruguay; 4 Analytical Biochemistry and Proteomics Unit, Institut Pasteur de Montevideo and Instituto de Investigaciones Biológicas Clemente Estable, Montevideo, Uruguay; 5 Human Molecular Genetics, INDICyO Program, Institut Pasteur de Montevideo, Montevideo, Uruguay; 6 Departamento de Histología y Embriología, Facultad de Medicina, Universidad de la República, Montevideo, Uruguay; 7 Area Biofísica, Departamento de Biología Celular y Molecular, Facultad de Veterinaria, Universidad de la República, Montevideo, Uruguay; 8 Department of Genomics, Instituto de Investigaciones Biológicas Clemente Estable, and Laboratory of Molecular Interactions, Facultad de Ciencias, Universidad de la República, Montevideo, Uruguay; 9 Departamento de Bioquímica and Centro de Investigaciones Biomédicas (CEINBIO), Facultad de Medicina, Universidad de la República, Montevideo, Uruguay; 10 Department of Nuclear Medicine and PET Centre, Aarhus University Hospital, Aarhus, Denmark; 11 Department of Biomedicine, Aarhus University, Aarhus, Denmark

**Keywords:** Biochemistry, Biochemistry, Tumour-suppressor proteins, Tumour-suppressor proteins, Tumour-suppressor proteins

## Abstract

The protein Deleted in Breast Cancer-1 is a regulator of several transcription factors and epigenetic regulators, including HDAC3, Rev-erb-alpha, PARP1 and SIRT1. It is well known that DBC1 regulates its targets, including SIRT1, by protein-protein interaction. However, little is known about how DBC1 biological activity is regulated. In this work, we show that in quiescent cells DBC1 is proteolytically cleaved, producing a protein (DN-DBC1) that misses the S1-like domain and no longer binds to SIRT1. DN-DBC1 is also found *in vivo* in mouse and human tissues. Interestingly, DN-DBC1 is cleared once quiescent cells re-enter to the cell cycle. Using a model of liver regeneration after partial hepatectomy, we found that DN-DBC1 is down-regulated *in vivo* during regeneration. In fact, WT mice show a decrease in SIRT1 activity during liver regeneration, coincidentally with DN-DBC1 downregulation and the appearance of full length DBC1. This effect on SIRT1 activity was not observed in DBC1 KO mice. Finally, we found that DBC1 KO mice have altered cell cycle progression and liver regeneration after partial hepatectomy, suggesting that DBC1/DN-DBC1 transitions play a role in normal cell cycle progression *in vivo* after cells leave quiescence. We propose that quiescent cells express DN-DBC1, which either replaces or coexist with the full-length protein, and that restoring of DBC1 is required for normal cell cycle progression *in vitro* and *in vivo*. Our results describe for the first time *in vivo* a naturally occurring form of DBC1, which does not bind SIRT1 and is dynamically regulated, thus contributing to redefine the knowledge about its function.

## Introduction

Deleted in Breast Cancer 1 (DBC1) is a nuclear protein with multiple roles in the regulation of cellular physiology^1^. It does so by binding and modulating the biological activity of several transcription factors and epigenetic regulators, including SIRT1^2–4^, HDAC3^5^, p53^6^, BRCA1^7^, AR^8^, ER^9,10^, Rev-erb-alpha^11^ and PARP1^12^, among others.

It is well documented that, in most cases, DBC1 binds to its targets through its N-terminal domain. This is the case for ER^10^, AR^8^, BRCA1^7^, SIRT1^2^, HDAC3^5^, SUV39H1^13^, and also the lncRNA MALAT1^14^. In the case of SIRT1 and other proteins, the N-terminal region on DBC1 responsible for binding was originally narrowed down to the LZ domain, which begins in the residue 243 in the human protein^1^. However, recent findings challenge this view^15^, suggesting that this regulation is more complex than previously believed. Moreover, to date information about how the interaction between DBC1 and its targets is regulated is scarce. We showed previously that in liver and fat tissue DBC1/SIRT1 interaction is regulated by metabolic load^4,16^. Later, we and other investigators showed that AMPK and PKA-dependent phosphorylation of SIRT1 regulated its binding to DBC1^17,18^. Also, it was shown that GAPDH phosphorylation by AMPK leads to SIRT1/DBC1 dissociation^19^. On the contrary, phosphorylation of DBC1 by ATM during genotoxic stress increases its binding and inhibition of SIRT1^20^. Besides the regulation of SIRT1/DBC1 association by phosphorylation, reports have shown that DBC1 binding to a given partner can be displaced by another DBC1 target^6,14,21^, suggesting that a stoichiometric balance among different proteins may play a role in target selectivity. However, since many of these results rely on overexpression experiments, more work should be done to strengthen their biological relevance

As its name indicates, DBC1 has been involved in cell cycle control and cancer. However, its role in cancer is not clearly defined, since some evidence suggests that DBC1 is tumorigenic, while others underscore a tumor suppressor function^1^. Nevertheless, it is well accepted that DBC1 plays a role in cell cycle control in many different cell lines, mainly through regulation of the proteins mentioned above. Of note however, most of the accumulated evidence has been obtained using transformed cells, where normal cell cycle control is typically altered. Indeed, up to date the role of DBC1 on cell cycle control *in vivo* or in non-transformed cells has not been studied in depth.

We and others have shown previously that DBC1 also plays a key role in the regulation of metabolism^4,11,16,18,22–24^. DBC1 binds to SIRT1 and inhibits its enzymatic activity during obesity^4,16^. Consistently, genetic deletion of DBC1 in mice protects against high-fat diet induced insulin resistance, non-alcoholic fatty liver disease^4^ and atherosclerosis^16^. In fat tissue, DBC1 regulates cellular senescence during obesity through modulation of HDAC3 activity^23^, whereas in the liver, DBC1 participates in the control of gluconeogenesis, regulating PEPCK activity^22^.

Unlike most tissues in mammals, the liver has a unique regeneration capacity. In a normal liver, hepatocytes are mostly in a quiescent state (G0) of the cell cycle. When the liver is injured or partially removed, hepatocytes massively exit G0 and undergo a series of synchronic rounds of division until the original organ size is recovered^25^. Once total regeneration has been achieved, hepatic cells go back into quiescence^25^, making liver regeneration a remarkable model to study cell cycle control *in vivo*, especially G0/G1 transitions. Importantly, understanding how the liver regeneration potential is regulated is key for the treatment of acute liver injury and liver transplant^26^. The mechanisms that regulate liver regeneration are not completely known and are of critical importance for understanding liver function in normal and pathological conditions. In this context, it is worth pointing out that SIRT1 has been involved in liver regeneration. A recent report using transgenic mice overexpressing SIRT1 showed impaired liver regeneration, compared to WT mice. Also, SIRT1 transgenic mice showed a delay in G0-G1/S transitions^27^.

The aim of this work was to gain insight into the mechanisms of regulation of DBC1 biological activity during the cell cycle *in vitro* and *in vivo*. We found that in quiescent cells, DBC1 is processed by proteolytic cleavage in the N-terminus, producing a novel form of the protein (DN-DBC1). We mapped the cleavage sequence of DN-DBC1 to the residues 124–138, meaning that DN-DBC1 loses the S1-like binding domain. In agreement with this, we found that DN-DBC1 does not bind to SIRT1, suggesting that DN-DBC1 is inactive in terms of SIRT1 regulation. Tissue analysis in mouse and human samples showed that DN-DBC1 can be found *in vivo*. Also, we show that DN-DBC1 is rapidly down-regulated when quiescent cells re-entered the cell cycle, findings that were also observed *in vivo* in a model of liver regeneration. Finally, we show that DBC1 KO mice show impaired liver regeneration after partial hepatectomy, suggesting that DBC1, and probably the dynamic regulation of the DN-DBC1/DBC1 ratio is necessary for correct cell cycle progression during liver regeneration.

In summary, our results provide evidence that the function of DBC1 is regulated during cell cycle *in vitro* and *in vivo*. We propose that during quiescence DBC1 is processed resulting in a N-deleted protein (DN-DBC1) that is unable to bind SIRT1. When cells re-enter the cell cycle, DN-DBC1 is rapidly replaced by DBC1. Our results suggest that downregulation of DN-DBC1 and replacement by DBC1 is necessary to inhibit SIRT1 during normal liver regeneration, an observation that is consistent with the data already reported. Altogether, our findings provide compelling new evidence regarding the regulation of DBC1 *in vivo*, and bring new insights to understand its role in liver regeneration and cell cycle control, findings that are potentially relevant to dissect the function of DBC1 in cancer.

## Materials and Methods

### Animal handling and experiments

All mice used in this study were maintained at the Institut Pasteur de Montevideo Animal facility (UATE). The experimental protocol was approved by the Institutional Animal Care and Use Committee of the Institut Pasteur de Montevideo (CEUA, Protocol number 009-16) and studies were performed according to the methods approved in the protocol. WT and whole-body DBC1 KO mice were in a C57BL6/J pure background. DBC1 KO mice were backcrossed into C57BL/6J for more than 10 generations in order to ensure genetic purity. Only male mice (8–14 weeks old) were used in this study. In all experiments, groups consisted of at least 3–4 mice per group.

### Partial hepatectomy and liver regeneration

To study liver regeneration, we performed 2/3 hepatectomy in mice as described previously^28^. Briefly, mice were weighed before surgery. Anesthesia was induced by isoflurane. Skin was disinfected with 70% ethanol and a midline incision in abdominal skin and muscle was made. The xiphoid apex was lifted upward and backward according to the instructions shown by the above-mentioned authors in the supplementary video on-line. The peritoneal cavity was held open by retractors. Once the liver was exposed it was manipulated by saline-moistened cotton tips. First, the left lateral lobe was completely ligated with a 4–0 silk thread and then cut. Afterwards, the median lobe was ligated not too close to the supra-hepatic vena cava in order to avoid venous obstruction and then cut as well. The remaining liver consisted of the right and caudate lobes. The peritoneum was closed with a 5–0 silk suture and the skin with wound clips. Buprenorphine hydrochloride was administered at the site of the incision for analgesia. The entire procedure lasted 15 minutes. Mice were placed on a warming pad for recovery. The dissected tissue was weighed. If acute bleeding occurred or if animals did not recover properly and showed signs of suffering, they were euthanized. Mice were controlled every 12 hours after hepatectomy.

### Human liver tissue

Liver tissue specimen was obtained from the lateral portion of a partial hepatectomy performed upon clinical indication. Informed consent to the procedure was given. Anonymized tissue was addressed in accordance with the Danish National Committee on Health Research Ethics guidelines on the use of biological material in health research projects, where approval from local Danish ethical committee is not required unless the research is related to artificial fertilization.

### Histology

Liver sections were obtained from paraffin-embedded tissue and stained with H&E following standard procedures. Liver cyto-architecture and quantitation of mitotic index were analyzed double blinded by independent personnel. For each mouse, at least 3 sections were analyzed.

### *In vivo* BrdU incorporation

Two hours before euthanasia, 5-Bromo-2′-deoxyuridine (BrdU, Sigma) was injected to mice i.p. (100 mg/kg). After that time, mice were deeply anesthetized with ketamine and xylazine. Blood was obtained with heparinized syringes and hepatic tissue from the right lobe was either frozen in liquid nitrogen for qPCR and western blot or fixed in 4% paraformaldehyde (PFA) for 24 hours for immunohistochemistry.

### RNA isolation and qPCR

Total RNA from livers was isolated with TRIzol by standard procedures. qPCR was carried out using Taqman probes as follows: Cyclin A2 (Mm00438063_m1), Cyclin E1 (Mm01266311_m1), Cyclin B1 (Mm03053893_gH), Cyclin D1 (Mm00432359_m1), Sirt1 (Mm01168521_m1). Expression analysis was calculated as fold increase with respect to control.

### Cell growth and maintenance

Mouse Embryonic Fibroblasts (MEFs) were obtained from E13-E14 embryos following standard procedures. IMR90 and HepG2 cells were obtained from ATCC. Cell growth and maintenance was performed in standard conditions in a humidified CO_2_ incubator at 37 °C and 5% CO_2_. Unless otherwise specified, cell culture medium was Dulbecco´s Modified Eagle Medium High Glucose (DMEM – Thermo Fisher Scientific, #61965026), supplemented with fetal bovine serum 10% (FBS), glutamine 2 mM, Hepes 10 mM, penicillin 10,000 U/mL and streptomycin 10,000 μg/mL (Thermo Fisher Scientific, #16000044, #25030081, #15630080, #15140122, respectively). Washes were performed with phosphate-buffered saline (PBS – Thermo Fisher Scientific, #14190094). Experiments in MEFs were performed between passages 2–5; experiments in IMR90 were performed between passages 5–15.

### Induction of cell cycle arrest

Cell cycle arrest was induced by serum deprivation in primary MEFs and HepG2, or contact inhibition (IMR90). Cell cycle arrest induced by serum deprivation was performed in cells initially cultured in standard conditions. Once cells reached 50–60% confluence they were washed twice with PBS and fresh serum-deprived medium was added. The serum concentrations and the period of time in which cells remained under conditions of serum-deprivation are specified in each experiment.

### Induction of cell senescence

Early passage IMR90 human fibroblasts (population doubling level 25) were transduced with lentivirus carrying a plasmid for expression of the oncogene Ras (pLenti CMV/TO RasV12 Puro, w119-1 was a gift from Eric Campeau, Addgene plasmid # 22262); or a lentivirus containing a control plasmid (Open Biosystems). Cells were selected with puromycin (2 μg/ml). Ras expression was verified by western blot. Senescence associated β-galactosidase (SA-β-Gal) activity was determined as described previously^29^. Percentage positive staining was calculated by counting at least 100 random cells in 5 different microscopic fields. Experiments were performed two weeks after infection.

### BrdU Incorporation in cells

To analyze BrdU-positive cells, 3 × 10^5^ MEFs, were plated in 5 plates/10 cm each (including coverslips), in DMEM supplemented with 10% FBS, 100 U/ml penicillin, 100 µg/ml streptomycin, 2 mM glutamine and 10 mM Hepes. Cells were synchronized by serum deprivation (0.1% FBS) for 48 hours and later incubated with 10% FBS for 6, 12, 24, 30 and 48 hours, and 2 hours before harvesting, they were incubated with 10 µM BrdU. Cells were counted in a Neubauer chamber. Coverslips were washed 3 times with PBS, fixed using 4%PFA × 15 minutes, incubated with 2 M HCl for 15 minutes at 37 °C, blocked with 2% BSA-0.03% Triton x-100 during 1 hour and then incubated with a rat anti-BrdU antibody (Abcam). Finally, a goat anti-rat Alexa Fluor 488 labeled antibody (Invitrogen) was used and samples were analyzed using an epifluorescence microscope (Olympus IX81).

### Protein extraction and immunoprecipitations

Protein extraction was performed using NETN lysis buffer (Tris-HCl pH 7.4 20 mM, NaCl 100 mM, EDTA 1 mM, IGEPAL 0.5% –Merck, #T1503, #S3014, #E6758, #I8896, respectively) supplemented with NaF 5 mM, Nicotinamide 5 mM, β-glycerophosphate 50 mM and protease inhibitor cocktail 1.4 mg/mL (Merck, #S6776, #N0632, #G9422 and #S8830, respectively). Cultured cells were first washed with PBS to remove cell debris and then collected with PBS using a cell scraper. Cells were pelleted, resuspended in lysis buffer (in a volume ratio of 1:10), and then incubated during 20–30 minutes at 4 °C under constant agitation. Lysates were then centrifuged at 10,000 g during 10 minutes at 4 °C and supernatants conserved; these represent the soluble protein extracts. Hepatic tissue was fast-frozen by incubation into liquid nitrogen immediately after its dissection. One-hundred mg of hepatic tissue were first cut into small pieces and further processed with lysis buffer (in a volume ratio of 1:10) and 0.5 mm zirconium-oxide beads (in a volume ratio of 1:2) using a Bullet Blender (Next Advance, #BBY24M). Velocity and time conditions were set as recommended by manufacturers. Homogenates were then incubated during 20–30 minutes at 4 °C under constant agitation. Lysates were then centrifuged at 10,000 g during 10 minutes at 4 °C and supernatants conserved; these represent the soluble protein extracts. Protein extracts used for immunoprecipitation assays were first pre-cleared by incubating 1 mg of total protein with 20 μL of Protein A/G PLUS-Agarose (Santa Cruz Biotechnology Inc. #SC-2003) during 30 minutes at 4 °C under constant agitation. The pre-cleared protein extracts were then incubated with 20 μL of Protein A/G PLUS-Agarose and 4 μg of anti-DBC1 (Bethyl, #A303-942) or a non-specific IgG (Merck, #I5006) during 1 hour at 4 °C under constant agitation.

### Western blots

Protein extracts and immunoprecipitation were resuspended in Laemmli 2X. SDS-PAGE and subsequent protein transfer to PVDF membrane, blocking and washes were performed at standard conditions. Primary antibodies utilized were anti-DBC1 (Bethyl, #A300-434 and #A300-432), anti-SIRT1 (Cell Signalling, #9475), anti-Cyclin D1 (Abcam, #ab137875), anti-β actin (Merck, #A5441), anti-α tubulin (Merck, #T6074), anti-GAPDH (Abcam, #ab9485 and Cell Signalling, #2118), anti-cyclin A2 (Abcam, #ab181591), anti-Ras (Cell Signalling, #3339) and anti-FLAG (Sigma, #F3165). Secondary antibodies utilized were HRP-conjugated IgG from rabbit, mouse and goat (Merck, #A0545, #A9044 and #A8919, respectively). Blot developing was performed with SuperSignal West Pico Chemiluminescent Kit (Pierce, #34080) and results were processed by densitometry analysis with ImageJ (Rasband, W.S., Bethesda, Maryland, USA).

### SIRT1 activity

SIRT1 activity was determined with a SIRT1 Fluorometric Kit (Enzo Life Sciences) according to the manufacturer’s instructions and as described previously by us^4,16,18,30^. Briefly, this assay uses a small lysine-acetylated peptide, corresponding to K382 of human p53, as a substrate. The lysine residue is deacetylated by SIRT1, and this process is dependent on the addition of exogenous NAD^+^. The fluorescence values obtained in the absence of NAD^+^ did not differ from the blank. Addition of exogenous NAD^+^ was necessary, most likely because endogenous NAD^+^ was lost during sample preparation. SIRT1 inhibitors nicotinamide (2 mM), suramin (100 μM), and sirtinol (100 μM) were used to confirm the specificity of the reaction. Samples were homogenized in NETN buffer as described above. Homogenates were then incubated for 10 minutes at 37 °C to allow degradation of any contaminant NAD^+^. Next, 10 mM DTT was added to the medium, and homogenates were incubated again for 10 minutes at 37 °C. The homogenates (20–30 μg protein/well) were then incubated in SIRT1 assay buffer in the presence of either 100 μM Fluor de Lys–SIRT1 substrate (Enzo Life Sciences) and 5 μM TSA to determine the SIRT1-independent activity, or with 100 μM Fluor de Lys–SIRT1 substrate, 5 μM TSA and 200 μM NAD^+^ to determine the SIRT1-dependent activity. After 1 hour of incubation at 37 °C, the reaction was terminated by adding a solution containing Fluor de Lys Developer (Enzo Life Sciences) and 2 mM nicotinamide. Plates were incubated at 37 °C for 1 hour. Values were determined on a fluorometric plate reader (Spectramax Gemini XPS; Molecular Devices) with an excitation wavelength of 360 nm and an emission wavelength of 460 nm. Calculation of net fluorescence included the subtraction of a blank consisting of buffer containing no NAD^+^ and expressed as a percentage of control. The SIRT1-dependent activity was calculated after subtracting fluorescence values obtained in the absence of NAD^+^. In all cases, we confirmed the linearity of the reaction over time.

### Mass spectrometry

Both DBC1 and DN-DBC1 proteins were purified by immunoprecipitation as described above. Immunoprecipitants were resuspended in Laemmli buffer 2X and run in an 8.5% SDS-PAGE under standard conditions. Gels were then stained by colloidal Coomassie Blue and selected bands were manually excised and processed for mass spectrometry analysis as previously described^31^. Briefly, in-gel digestion was performed overnight at 37 °C by incubation with trypsin (sequencing grade, Promega); the resulting peptides were extracted with aqueous 60% acetonitrile containing 0.1% trifluoroacetic acid (TFA), and vacuum-dried. Tryptic peptides were desalted using C18 micro-columns (OMIX pipette tips, Agilent) and eluted with matrix solution (saturated solution of α-cyano-4-hydroxycinnamic acid in 60% acetonitrile 0.1% TFA) directly onto the MALDI sample plate. Mass spectra were acquired in a 4800 MALDI TOF/TOF instrument (Abi Sciex) in positive reflector mode and were externally calibrated using a mixture of standard peptides (Applied Biosystems). Selected peptides were further analyzed by MS/MS. Protein identification was performed by searching SwissProt database (2017_09; 555594 sequences) using the MASCOT program (Matrix Science http://www.matrixscience.com/search form select.html). The following search parameters were used: monoisotopic mass tolerance, 0.05 Da; fragment mass tolerance, 0.6 Da; methionine oxidation and cysteine carbamidomethylation as variable modifications; and one missed tryptic cleavage allowed. Protein mass and taxonomy were unrestricted. Significant protein scores (p < 0.05), and at least one peptide ion significant score (p < 0.05) per protein were used as criteria for positive identification. The differential signals in the spectra of the analyzed protein bands were fragmented and their sequence assignments were further corroborated by manual inspection of the MS/MS spectra.

### Plasma transaminases quantification

The extent of liver injury at different time points after hepatectomy was evaluated by quantification of aspartate aminotransferase (AST) and alanine aminotransferase (ALT) in plasma. Blood was obtained from anesthetized mice with heparinized syringes and then spinned at 10,000 g for 5 minutes at 4 °C to separate plasma from blood cells. Plasma AST and ALT levels were measured by GOT (AST) or GPT (ALT) IFCC modified liquid UV test (Human).

### Statistics

Values are presented as mean ± SEM of 3–5 experiments, unless otherwise indicated. For liver regeneration experiments, the results were analyzed by Two-way ANOVA. In all cases at least 3–4 animals per experimental condition were used. The exact number of mice in each experiment is specified in the corresponding figure legends. When comparing two different experimental conditions, 2-tailed Student’s *t* test was used. A *P* value less than 0.05 was considered significant.

## Results

### Quiescent cells express a low molecular weight form of DBC1 that lacks the N-terminal region

**Figure 1:**
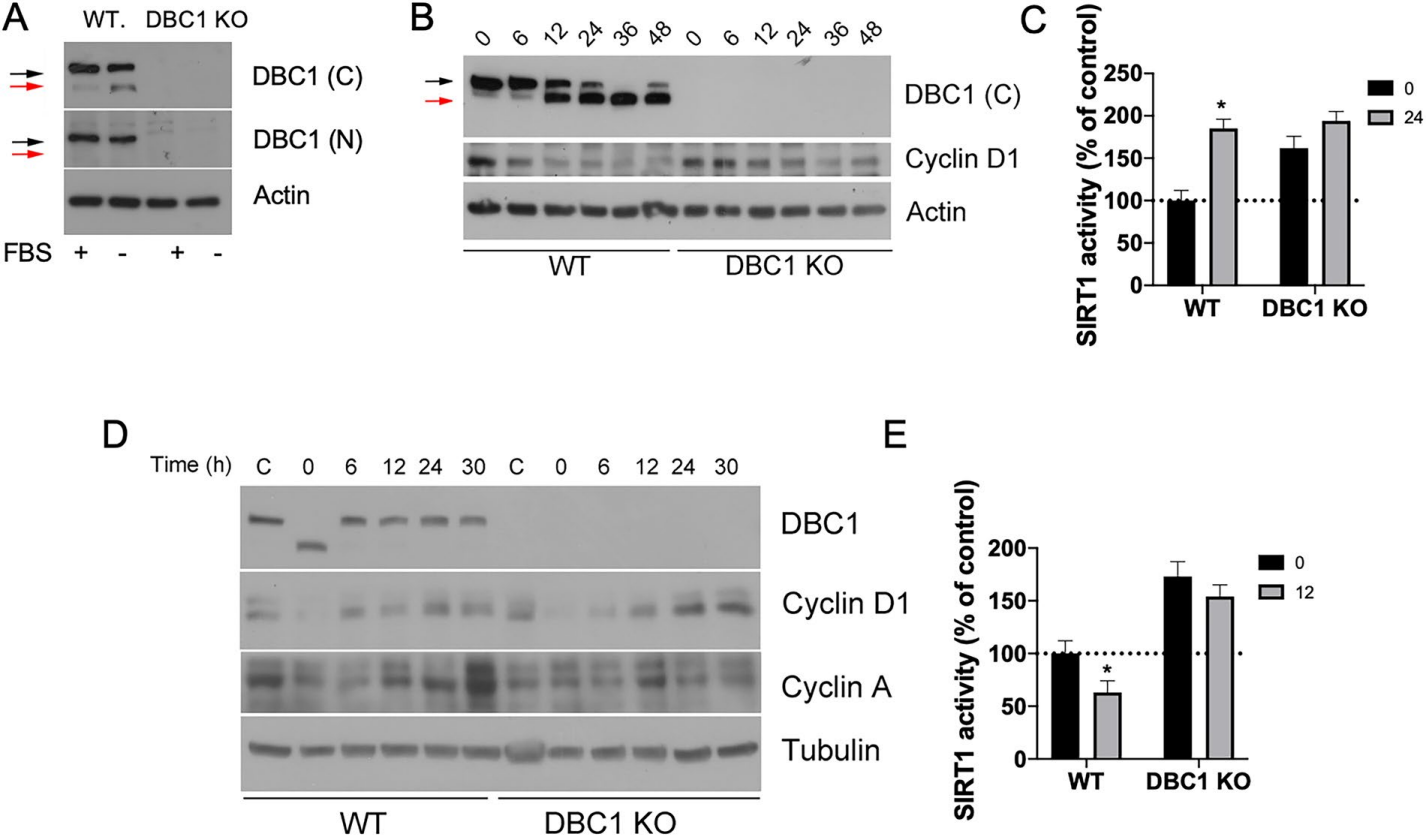
Quiescent cells dynamically express a low molecular weight form of DBC1 that lacks the N-terminal region. (**A**) WT and DBC1 KO MEFS were maintained either in 10% FBS (+) or in Serum free media (−) for 48 hours. Western blot for DBC1 was performed using an antibody for the C-terminal domain (sequence surrounding aa 875) or an antibody for the N-terminal domain (aa 1–50). Black arrows point to DBC1. Red arrows show the lower molecular weight band. (**B**) WT MEFS were incubated in serum-free media for different times. (**C**) SIRT1 activity was measured in WT and DBC1 KO MEFs at time 0 or after 24 hours of serum deprivation. *means p < 0.05 compared to Control (t0) for each genotype, unpaired t-test. (**D**) WT and DBC1 KO MEFS were incubated either in the presence of 10% FBS (**C**) or in serum-free media (−) for 48 hours. At time 0, media was replaced for 10% FBS (+) in quiescent cells and cells were collected at different time points. (**E**) SIRT1 activity was measured in WT and DBC1 KO MEFs at time 0 (48 hours of serum deprivation) or after 12 hours of serum (10% FBS) replenishment. *means p < 0.05 compared to Control (t0) for each genotype, unpaired t-test.

We have shown previously that DBC1 activity is regulated by energy status, both *in vitro* and *in vivo*^4^. In fact, we showed that energy deprivation abolished DBC1 binding to SIRT1 in hepatic cells^4^. However, the mechanisms that regulate this interaction remain to be elucidated. In order to gain knowledge into this regulation, we exposed MEFs to serum deprivation, which induces cellular quiescence (G0 synchronization). We found that in primary MEFs, induction of cell cycle arrest by serum deprivation led to the appearance of a lower molecular weight band of DBC1. This band became apparent only when using an antibody against the C-terminus of DBC1, but not against the N-terminus (aa 1–50) and it was not present in DBC1 KO MEFs (Fig. 1A). This short form of DBC1 (DN-DBC1) became apparent between 6–12 hours after serum removal, and it remained present for as long as cells were in quiescence (Fig. 1B). The replacement of DBC1 by DN-DBC1 in response to serum deprivation was accompanied by an increase in SIRT1 activity in WT MEFs (Fig. 1C). DBC1 KO MEFs showed increased SIRT1 activity in control conditions, but there was no further activation after serum deprivation (Fig. 1C). Interestingly, DN-DBC1 was rapidly down-regulated when serum was restored and replaced by full length DBC1 at the same time that cells re-entered into cell cycle, as can be seen by up-regulation of cyclins (Fig. 1D). Both cyclin D1 and A2 were downregulated during serum deprivation, coincidentally with the appearance of DN-DBC1. Upon serum restoring, DN-DBC1 was rapidly (6 hours) replaced by DBC1 coincidentally with up-regulation of cyclin D1 and before up-regulation of cyclin A2 (Fig. 1D), which typically peaks at the S/G2 transition. Taken together, these data suggest that DN-DBC1 is replaced by DBC1 early after cells re-enter into cell cycle, probably on G1 phase. Interestingly, replacement of DN-DBC1 by DBC1 after serum restoration was paralleled by an inhibition of SIRT1 activity in WT MEFs but not in DBC1 KO MEFs (Fig. 1E).

We also studied if DN-DBC1 is produced in response to another type of cell cycle arrest different from quiescence. For that, we induced cellular senescence by overexpression of the RAS oncogene in human cells (Supplementary Fig. 1). Interestingly, we found that DN-DBC1 was also produced in human quiescent cells, but not in RAS-induced senescent cells (Supplementary Fig. 1). Since it was proposed previously that DBC1 could be proteolytically processed during apoptosis^32,33^, we incubated resting cells with etoposide. In contrast, we did not observe DN-DBC1 after induction of apoptosis in quiescent cells (Supplementary Fig. 1).

### DN-DBC1 is a product of proteolytic cleavage of DBC1 and is also present *in vivo* in the liver

**Figure 2:**
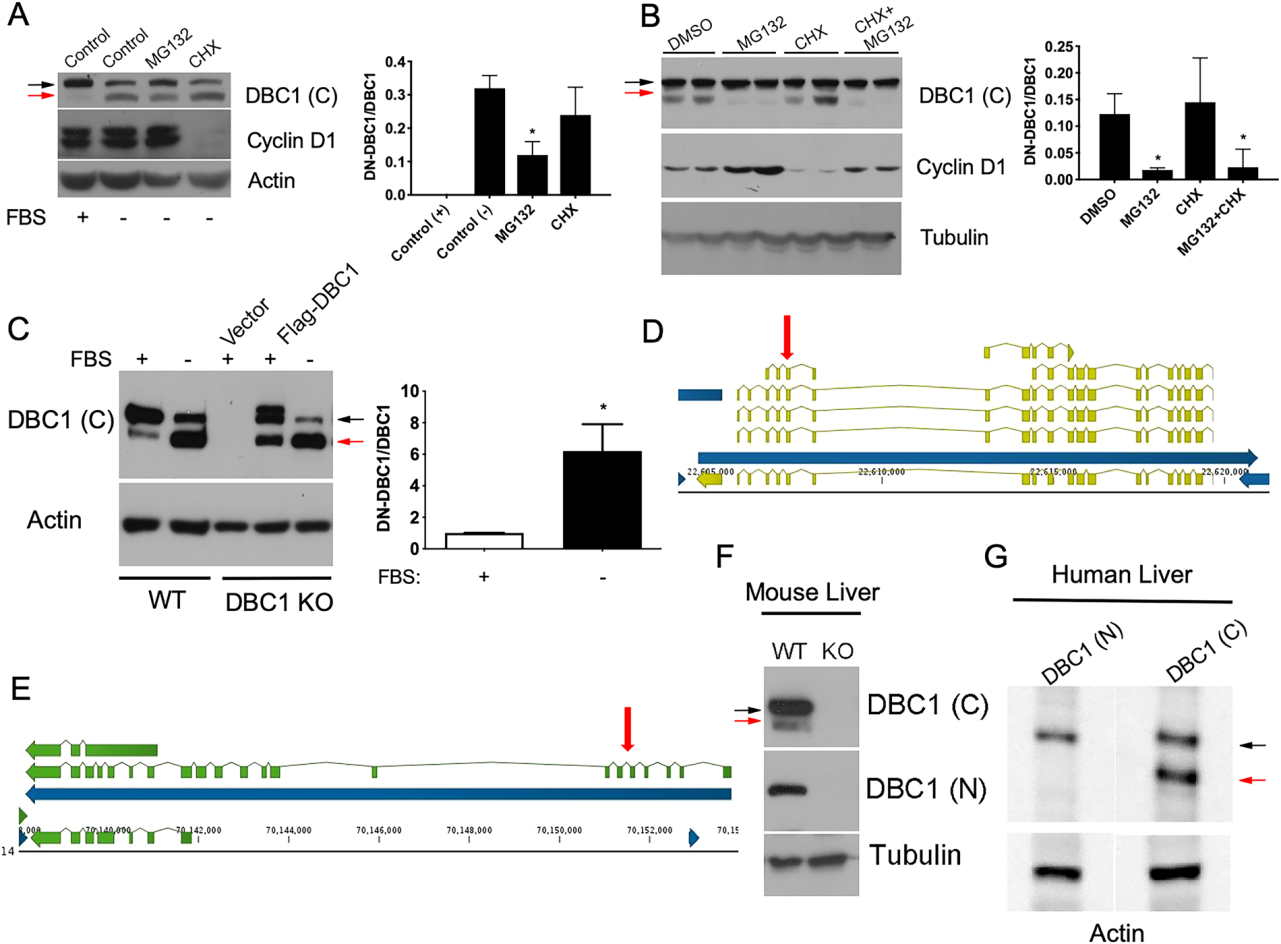
DN-DBC1 is a product of proteolytic cleavage of DBC1 and is also present *in vivo* in the liver. (**A**,**B**) WT primary MEFs (**A**) and IMR90 cells (**B**) were incubated in the presence (+) and absence (−) of FBS for 48 hours, an later with or without 10 μM MG132 or 100 μg/ml Cicloheximide (CHX) for 6 hours. Left, representative western blot. Right panel, quantitation of DN-DBC1/DBC1 ratio in three independent experiments. *means p < 0.05 compared to Control (−), One-way ANOVA. (**C**) DBC1 KO primary MEFs were transfected with empty vector or Flag-DBC1 vector and later incubated with (+) or without (−) FBS for 48 hours. WT MEFs were used as control. Left, representative western blot. Right, quantitation of Flag-DN-DBC1 in KO MEFs in three independent experiments. (**D**,**E**) To corroborate annotation and the number of possible splicing variants de novo transcript assembly and in silico translation was performed using transcriptomic data from human (**D**) and mouse (**E**) livers. For human transcript, *de novo* assembly the CHESS database was used. Green annotations summarize three possible transcripts from mice liver RNA-seq data. From top to bottom, the first and third were not previously annotated, the second matches with previous annotations. None of them when translated yielded the molecular weights observed in the present manuscript. E) depicts annotations in the case of human transcripts. Here, we can observe more splicing variants (several of them matched previous annotations) but *in silico* translation did not yield the observed molecular weights for DBC1 detected by the approaches used in this manuscript. Vertical red arrows show the exon where DN-DBC1 sequence begins. **F)** Representative western blot of liver samples isolated form WT and DBC1 KO mice using antibodies against the C-terminal (C) or N-terminal (N) regions. (**G**) Representative western blot of healthy human liver tissue using antibodies against the C-terminal (C) or N-terminal (N) regions.

Next, we investigated the mechanism of generation of DN-DBC1 in quiescent cells. Incubation of quiescent WT MEFs with the proteasome inhibitor MG132 partially restored DBC1 levels with a concomitant decrease in DN-DBC1 (Fig. 2A), suggesting that loss of the N-terminus domain is dependent on proteasomal activity and occurs at the expense of pre-existing DBC1. Similar results were obtained in human cells (Fig. 2B). In order to rule out *de novo* synthesis of DN-DBC1 during quiescence, we incubated quiescent cells in the presence of the protein synthesis inhibitor Cycloheximide (CHX) during the same time used with MG132, and found that DN-DBC1 was still produced both in human and mouse cells, even though protein synthesis was blocked, as evidenced by downregulation of cyclin D1 expression (Fig. 2B). To further confirm that DN-DBC1 was neither a novel alternative splicing product nor a variant not previously detected and reported in current genome annotations, we first transfected DBC1 KO MEFs with the cDNA coding for DBC1. Serum deprivation under these conditions led to an increase in the appearance of DN-DBC1 (Fig. 2C). Secondly, we analyzed sequencing data from normal mice liver transcriptome^34^. All splicing isoforms detected from mice did not produce, when translated *in silico*, products of the molecular weight of DN-DBC1. In fact, all splicing forms of DBC1 that contain an open reading frame included the N-terminus of the protein (Fig. 2D). This assessment was extended using annotations derived from the *de novo* assembly of transcripts performed previously from 10,000 human RNA-seq experiments gathered at the Chess database (See Supplementary methods for detailed information). Human DBC1 splicing variants were more abundant than the ones found in mice but none of them, when translated *in silico*^35^, yielded products comparable to DN-DBC1 (Fig. 2E). Altogether, these results show that DN-DBC1 is produced as a product of a proteasome-dependent proteolytic cleavage of pre-existing DBC1. Finally, we evaluated if DN-DBC1 is produced *in vivo* in mice and also in humans. To do this, we chose the liver, where most cells are in a quiescent state. We found that DN-DBC1 is expressed *in vivo* under normal conditions (Fig. 2F–G).

### DN-DBC1 lacks the S1-like binding domain and does not bind SIRT1

**Figure 3:**
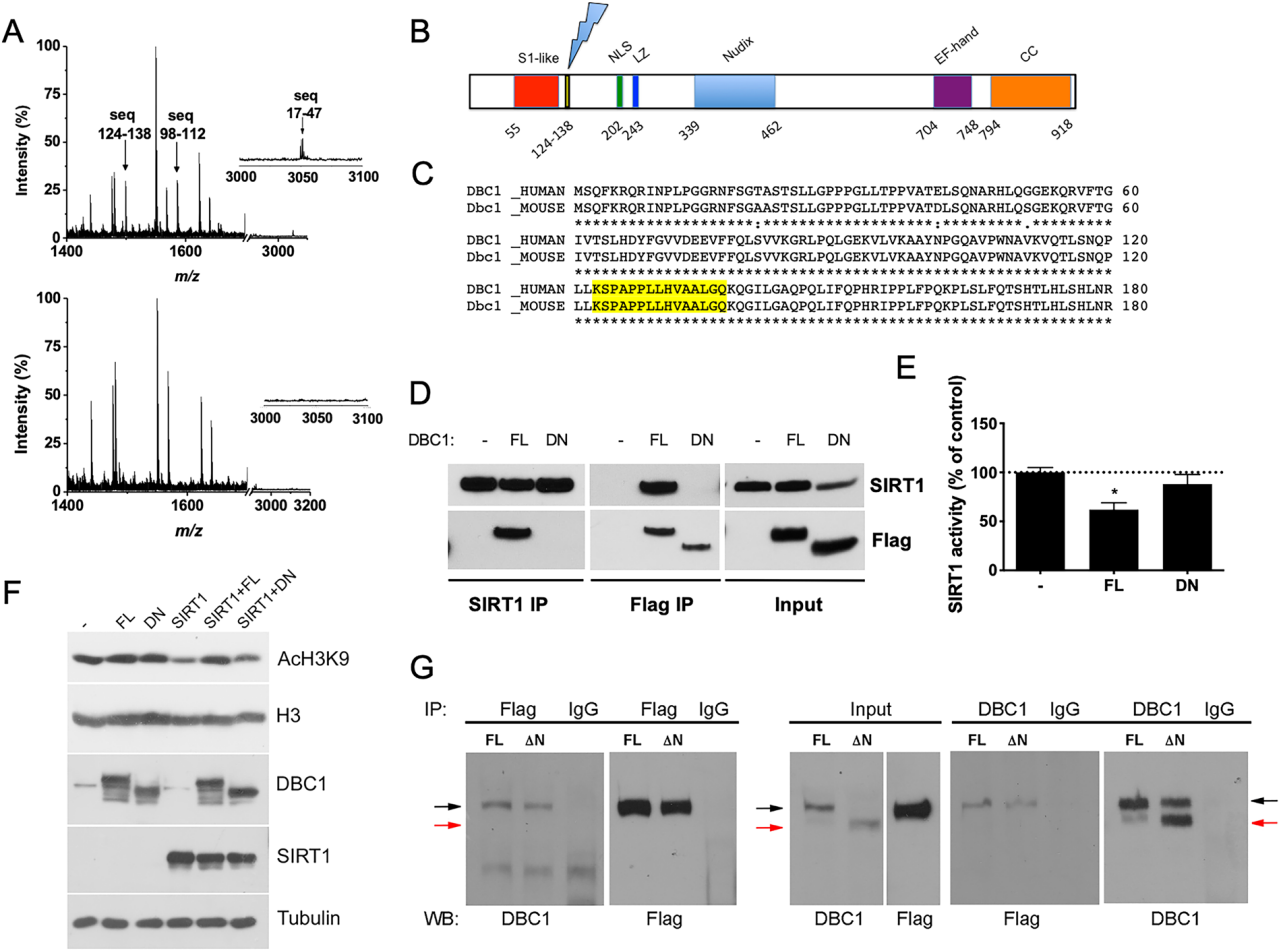
DN-DBC1 lacks the first 124–138 residues and maintains the LZ domain while not binding to SIRT1. (**A**) DBC1 (upper panel) and DN-DBC1 (lower panel) were purified by immunoprecipitation from mice livers and digested with trypsin previous to the analysis by mass spectrometry (MS). Upper panel, partial Mass Spectrometry (MS) spectrum of Full-length (FL) protein. Lower panel, partial MS spectrum of DN protein. Arrow in inset show the peak corresponding to peptides that were not detected in DN-DBC1 (**B**) Scheme of DBC1 sequence showing the different domains and the proposed site of cleavage. (**C**) Alignment of the first 188 amino acids of mouse and human DBC1. The region found to be lost in DN-DBC1 is highlighted in yellow, showing 100% aminoacidic residues conservation among species. (**D**) Flag-tagged FL and DN-DBC1 were overexpressed in 293 T cells and their binding to endogenous SIRT1 was evaluated by co-immunoprecipitation. Non-transfected cells were used as controls. A representative experiment out of three independent experiments is shown. (**E**) SIRT1 activity in 293 T cells transfected with empty vector (−) Flag-tagged FL, or Flag-tagged DN-DBC1. Activity was expressed as a percent of control (−) cells. *means p < 0.05 compared to control (−). One-way ANOVA. (**F**) Flag-tagged FL, DN-DBC1 and SIRT1 were overexpressed in 293 T. SIRT1 activity regulation was evaluated by H3 acetylation in Lysine 9 (AcH3K9). A representative western blot is shown. (**G**) SIRT1 and DBC1 interaction assayed by pull-down experiments. Flag-tagged recombinant SIRT1 was purified from overexpression in 293 T cells. 293 T cell lysates were incubated with liver lysates mainly expressing DBC1 (FL) or DN-DBC1 (DN). Pull-down assays were performed by incubating with Flag antibody (left panels) or DBC1 (C-terminus) antibody (right panels). Input is shown in the center. A representative experiment out of three independent ones is shown. Black and red arrows point to DBC1 and DN-DBC1 respectively.

Next, we mapped the region that is lost in DN-DBC1 compared to the full-length protein. We purified both DBC1 and DN-DBC1 from mouse liver by immunoprecipitation and analyzed them by mass spectrometry. We found that DN-DBC1 lacks the first 124–138 residues (Fig. 3A). Therefore, these data indicate that DN-DBC1 loses the S1-like binding domain while maintains the NLS and LZ domains (Fig. 3B). Sequence alignment between mouse and human DBC1 showed that the sequence mapped is 100% conserved among species (Fig. 3C). Next, we investigated if loss of the S1-like domain of DN-DBC1 affects its binding to one of DBC1 main targets: SIRT1. For that, we expressed a recombinant DN-DBC1 protein lacking the first 138 residues in 293 T cells and evaluated its binding to endogenous SIRT1 by co-immunoprecipitation. We found that the recombinant DN-DBC1 does not bind SIRT1, suggesting that this is an inactive form of the protein in terms of SIRT1 regulation (Fig. 3D). These results were further confirmed by measuring SIRT1 activity *in vitro*. Overexpression of FL DBC1 inhibit SIRT1 activity, while there was no inhibition when DN-DBC1 was overexpressed (Fig. 3E). We also measured *in situ* SIRT1 activity in 293 T cells by H3 acetylation in Lysine 9 (AcH3K9), a well-known target of SIRT1^36^. SIRT1 overexpression in 293 T cells decreased H3K9 acetylation (Fig. 3F). This effect was inhibited by overexpression of DBC1. However, overexpression of DN-DBC1 had no effect on SIRT1-dependent H3K9 acetylation (Fig. 3F). We also measured SIRT1 binding to endogenous DBC1 (full length) and DN-DBC1 by pull-down assays in livers. We incubated liver lysates with predominance of either DBC1 or DN-DBC1 (Fig. 3G, Input) with recombinant purified SIRT1 (Flag-SIRT1) and performed pull-down assays. When performing immunoprecipitation using Flag-SIRT1 and liver lysates, only DBC1 could be detected (Fig. 3G, left) in Flag immunoprecipitates, but not DN-DBC1. Similar results were obtained when DBC1 was immunoprecipitated with a C-terminus antibody. When DN-DBC1 was predominant in the livers, we found a decrease in Flag-SIRT1 binding (Fig. 3G, right). Altogether, these results suggest that endogenous DN-DBC1 does not bind to SIRT1, losing its SIRT1-inhibitory capacity

### The ratio between DN-DBC1 and DBC1 is dynamically regulated *in vivo* during liver regeneration, affecting SIRT1 function

**Figure 4:**
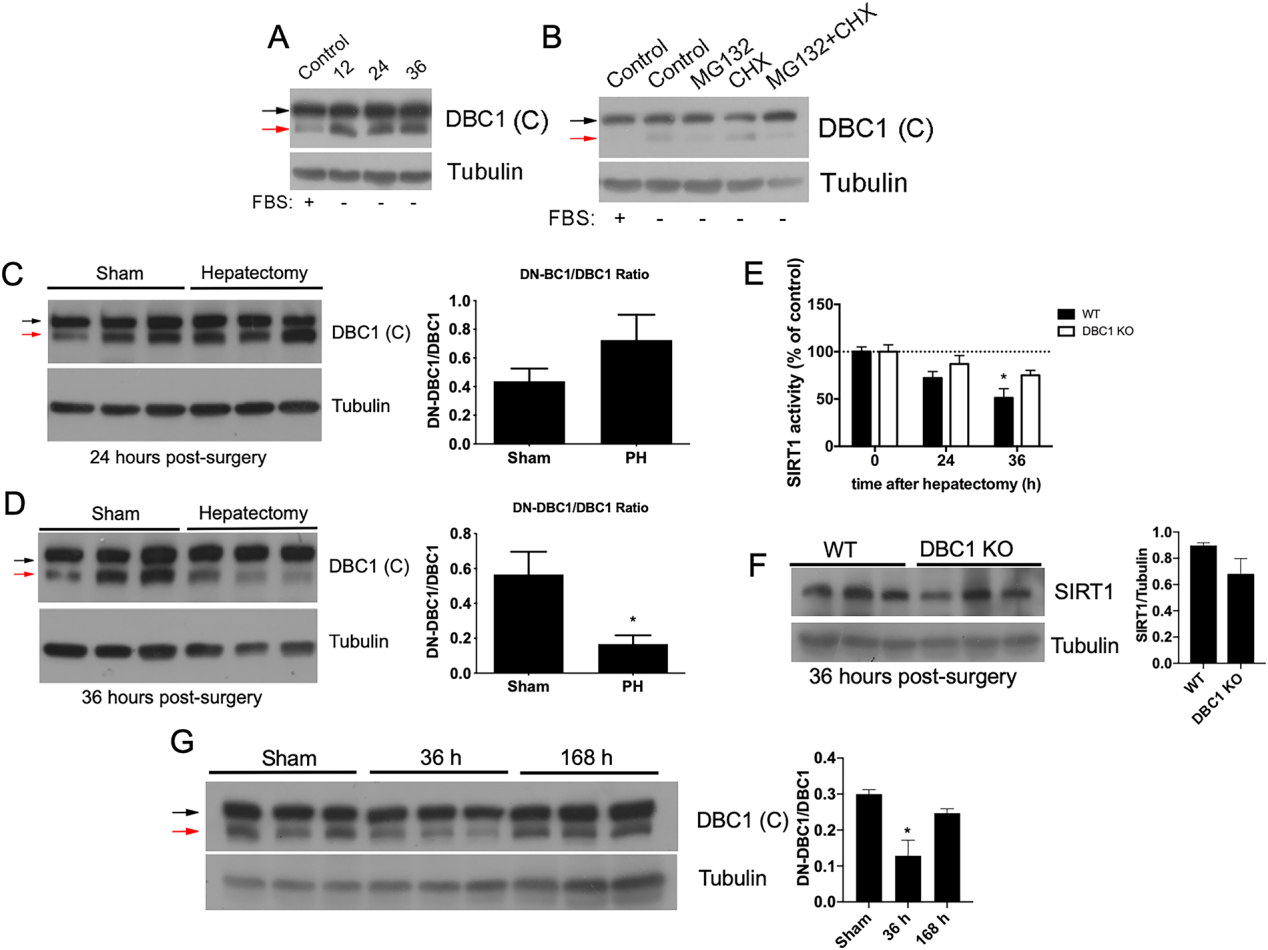
The ratio between DN-DBC1 and DBC1 is dynamically regulated *in vivo* during liver regeneration, affecting SIRT1 function. (**A**) Human hepatocytes (HepG2) were incubated in serum free media for 12, 24 and 36 hours. (**B**) HepG2 cells were incubated in the presence (+) and absence of FBS (−) for 24 hours, and later with or without 10 μM MG132 or 100 μg/ml Cycloheximide (CHX) for 6 hours. (**C**,**D**) Expression of DBC1 and DN-DBC1 in sham mice and 24 (C) or 36 (D) hours after partial hepatectomy (PH). Left, representative western blots. Right, quantitation of DN-DBC1/DBC1 ratio by densitometric analysis. *means p < 0.05, t-test. (**E**) SIRT1 activity was measured in isolated liver nuclei form WT and DBC1 KO mice in sham mice and 24 or 36 hours after partial hepatectomy. Activity was normalized to sham control condition in each genotype. DBC1 KO mice showed increased basal SIRT1 activity (See supplementary information). SIRT1 activity showed significant inhibition in WT mice 36 hours after hepatectomy compared to sham WT mice. *means p < 0.05. Two-way ANOVA. (**F**) SIRT1 levels were measured by western blot in WT and DBC1 KO mice 36 hours after partial hepatectomy. Left, representative western blot. Right, quantitation by western blot densitometry, (**G**) Representative western blot of DN-DBC1 and DBC1 after 7 days (168 hours) of partial hepatectomy. Left, representative western blot. Right, quantitation by densitometric analysis. *means p < 0.05, Two-way ANOVA.

In order to study the dynamics of DBC1/DN-DBC1 during cell cycle *in vivo*, we used a well-characterized model of 2/3 partial hepatectomy^28^, where hepatocytes re-enter massively form G0 into G1 after surgery. First, we confirmed that DBC1 and DN-DBC1 are dynamically regulated in hepatic cells. HepG2 cells showed a similar pattern of dynamic DBC1 regulation to fibroblasts. DN-DBC1 accumulated during quiescence (Fig. 4A). Also, the appearance of DN-DBC1 was regulated by MG132 but not by Cycloheximide (Fig. 4B), showing that DBC1/DN-DBC1 is regulated also in hepatic cells. Next, we performed 2/3 partial hepatectomy in WT mice and followed DBC1/DN-DBC1 dynamics by western blot. We found that 24 hours after surgery, DN-DBC1 was still present in hepatectomized mice, similar to sham mice (Fig. 4C). However, 36 hours after surgery there was a clear decrease in DN-DBC1 (Fig. 4D). In order to determine if changes in DN-DBC1/DBC1 dynamics during liver regeneration affect SIRT1 function, we measured its enzymatic activity in WT and DBC1 KO mice during liver regeneration. In sham mice, SIRT1 activity was higher in DBC1 KO compared to WT mice (Supplementary Fig. S2). When we performed liver surgeries in these mice, we found an inhibition of SIRT1 activity 36 hours after partial hepatectomy (Fig. 4E), although total SIRT1 levels remained unchanged (Fig. 4F and Supplementary Fig. S2). Interestingly, the inhibition of SIRT1 activity was coincidental with the decrease in DN-DBC1 levels (Fig. 4G) and was not observed in DBC1 KO mice (Fig. 4E). This suggests that replacement of DN-DBC1 by DBC1 plays a role in the inhibition of SIRT1 activity observed during regeneration. Finally, we measured DN-DBC1/DBC1 ratio 7 days (168 hours) after partial hepatectomy and found that by the time liver regeneration had achieved its end, DN-DBC1 was expressed to similar levels as sham mice (Fig. 4G).

### Absence of DBC1 delays liver regeneration by affecting normal cell cycle progression after partial hepatectomy

**Figure 5:**
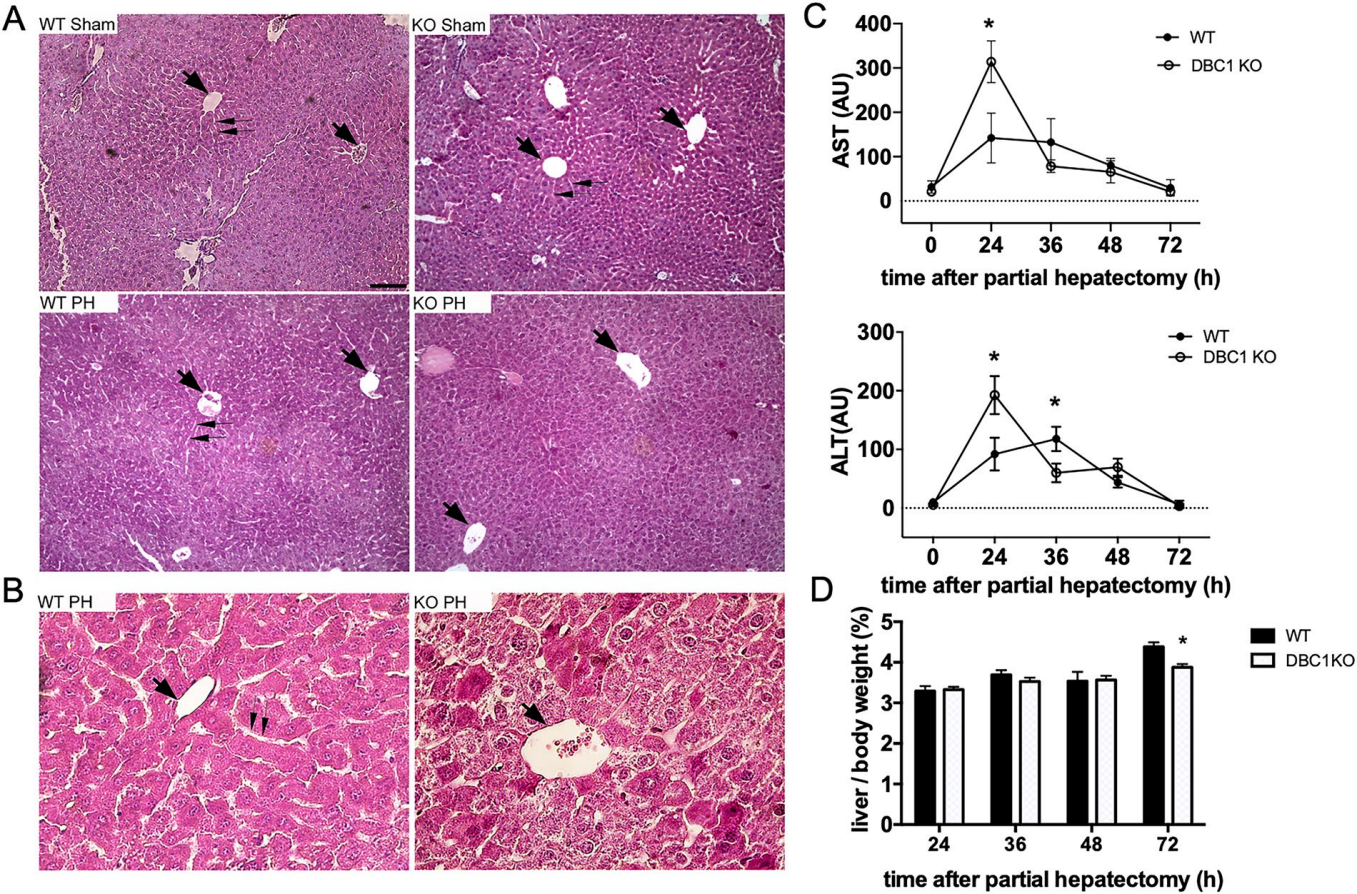
DBC1 KO mice have impaired liver regeneration after partial hepatectomy. (**A**,**B**) The cyto-architecture of control (sham) and regenerating livers (PH) differ between WT and DBC1 KO mice. (**A**) Lobule organization seems to be similar in intact (Sham) livers of WT and KO mice. However, after 72 hours of partial hepatectomy (PH) the lobule organization around the central vein (thick arrows) is almost complete in WT livers. Note that sinusoids (thin arrows) that are being formed in WT livers, are difficult to trace in lobules of DBC1 KO animals. Bar is 200 micrometers. (**B**) Higher magnification of livers after 72 hours of partial hepatectomy, showing a lobular central vein (thick arrow) and hepatocytes surrounding it to better appreciate the presence of newly formed sinusoids (thin arrows) in WT livers and their absence in KO ones. Bar is 50 micrometers. (**C**) Aspartate aminotransferase (AST, upper panel) and Alanine aminotransferase (ALT, lower panel) activity measured in plasma of WT and DBC1 KO mice during liver regeneration. *p < 0.05, two-way ANOVA (**D**) Time course of liver mass recovery after partial hepatectomy in WT and DBC1 KO mice. Liver mass was normalized to total body weight. *p < 0.05 Unpaired t-test at each time point. Total liver mass was not different between WT and DBC1 KO in sham control mice.

**Figure 6:**
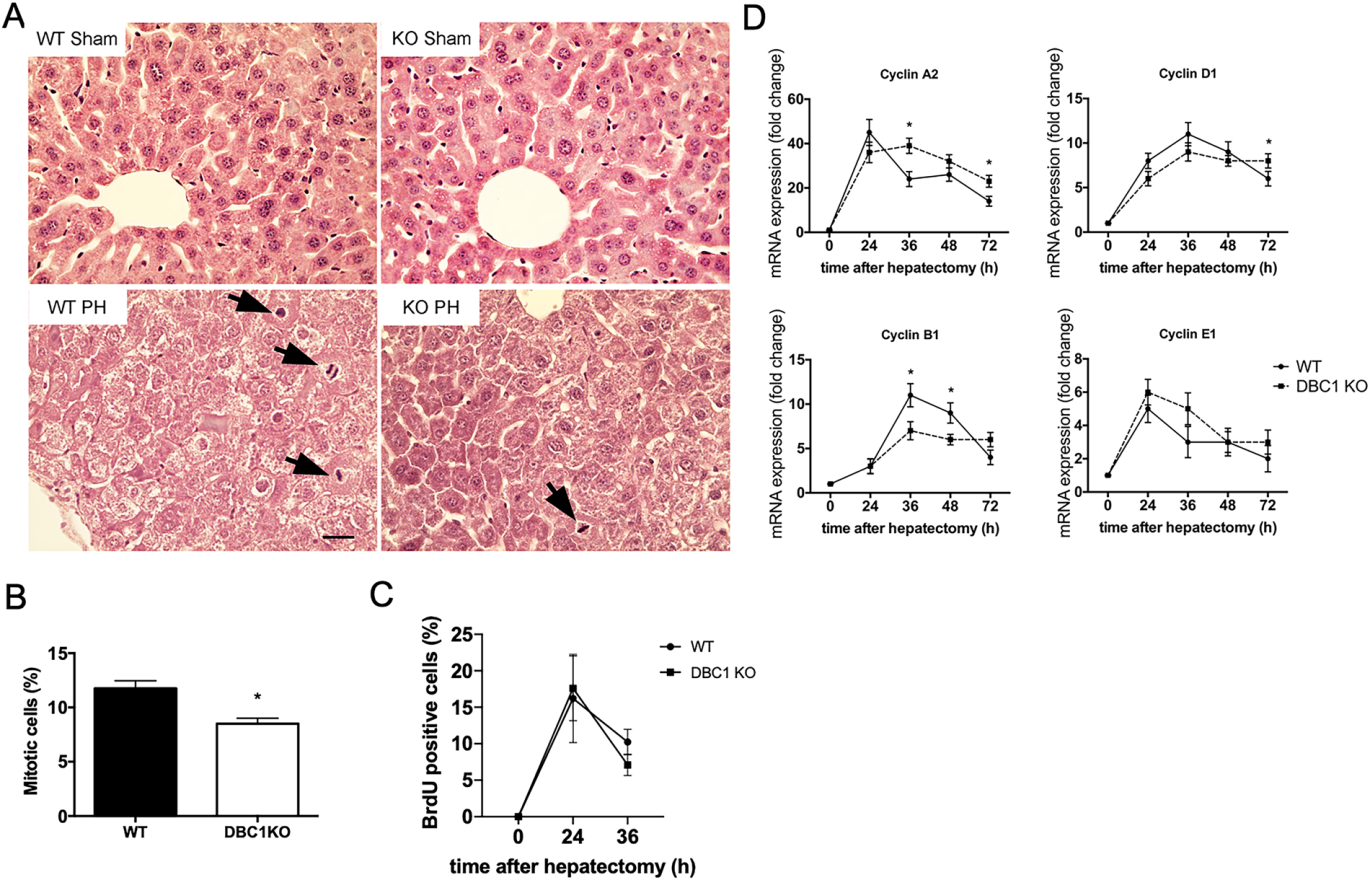
Absence of DBC1 impairs cell cycle progression after cells leave quiescence *in vivo*. (**A**) Representative H&E pictures of sham (upper panels) and regenerating (PH, lower panels) livers obtained from WT (left panels) and DBC1 KO mice (right panels) 36 hours after partial hepatectomy. Arrowheads show cells undergoing mitosis. (**B**) Quantitation of percent of mitotic cells. Mitotic cells were quantified in double-blinded experiments from H&E-stained sections. *p < 0.05, two tailed unpaired t-test. (**C**) Quantitation of BrdU incorporation into livers of WT and DBC1 KO mice at 0, 24, and 36 hours hours after partial hepatectomy. BrdU (100 mg/kg) was injected i.p. 2 hours before euthanasia. BrdU positive cells were quantified as percent of total cells. (**D**) Time course of cell cycle progression markers from WT and DBC1 KO livers during regeneration. *p < 0.05, Two-way ANOVA between groups.

A previous report showed that transgenic liver-specific SIRT1-overexpressing mice have impaired liver regeneration^27^. Based on this study, and our data suggesting that SIRT1 activity is regulated by DBC1/DN-DBC1 during liver regeneration, we compared the liver regeneration capacity in WT and DBC1 KO mice during the first 72 hours after partial hepatectomy. First, we studied liver architecture during regeneration in WT and DBC1 KO mice. The evaluation of H&E stained slices of livers undergoing regeneration revealed histological differences between genotypes. After 72 hours of hepatectomy, the livers of WT mice showed a histological organization of liver lobules reminiscent of those present in the mature intact organ: a clearly identifiable central vein (Fig. 5A,B, left panels; thick arrows) surrounded by differentiated hepatocytes arranged close to sinusoids (Fig. 5A,B, left panels; thin arrows). In contrast, after 72 hours of hepatectomy the DBC1 KO mice livers showed a less structured pattern of organization of the hepatic lobule, almost lacking sinusoids (Fig. 5A,B, right panels). We also found that AST and ALT levels 24 hours after the hepatectomy were significantly higher in DBC1 KO mice compared to WT animals (Fig. 5C), although this difference was lost later during regeneration. We also followed mass liver recovery over time after the surgery, and found that DBC1 KO mice have a delay in liver mass recovery after the surgery (Fig. 5D). Moreover, when we measured liver mass recovery one week after surgery, we found that WT mice fully recovered the original mass, but total liver mass was still lower in the DBC1 KO (Supplementary Fig. S2). Despite this, there was no difference in survival between WT and DBC1 KO mice after surgery (Supplementary Fig. S2). Next, we assessed cell division in WT and DBC1 KO regenerating livers. First, we analyzed liver tissue for mitotic cells 36 hours post-surgery, and we found that livers from DBC1 KO mice showed significantly less mitotic cells (Fig. 6A, arrows and Fig. 6B), suggesting DBC1 KO cells have altered cell proliferation after surgery. However, we measured BrdU incorporation in the regenerating livers from WT and DBC1 KO mice and found no difference between genotypes (Fig. 6C), suggesting that DBC1 KO mice have no compromise in the early response to the liver surgery and cell cycle entry.

Analysis of the expression of different mRNAs coding for cyclins confirmed that DBC1 KO mice had alterations in normal cell cycle progression compared to WT mice. Cyclins showed no significant difference in expression in the early phases of liver regeneration. Moreover, both WT and DBC1 KO mice cyclins A2, D1, and E1 were all up-regulated in the first 24 hours of liver regeneration. However, later on during regeneration, the expression pattern of cyclins started to differ. Cyclin A2 expression became statistically different at 36 hours post-surgery, being higher in the DBC1 KO mice, and remaining higher for as long as 72 hours post-surgery (Fig. 6D). On the other hand, Cyclin D1 levels in WT livers decreased after 72 hours, time where livers were already showing liver mass recovery, while in the DBC1 KO mice failed to go down (Fig. 6D). Cyclin B1, which is normally up-regulated prior to mitosis, peaked at 36 hours post hepatectomy in WT mice, with its expression decreasing afterwards. DBC1 KO mice however failed to show a peak at that time. There were no significant differences in Cyclin E1 expression along the time course of the experiment (Fig. 6D). This data suggested that early phases of liver regeneration start normally in DBC1 KO mice, but there might be a later impairment in the normal process of reconstitution of liver mass. Finally, we studied cell cycle progression and duplication in WT and DBC1 KO MEFs after G0 release. We found that similar to what we observed *in vivo*, DBC1 KO MEFs showed a delay in cell division rate upon G0 release (Supplementary Fig. S3), suggesting that restoring of full length DBC1 is necessary for normal cell cycle progression when cells move from a quiescent into an actively dividing state both *in vitro* and *in vivo*.

## Discussion

In this study, we show that DBC1 can exist in two different forms, the full length DBC1 and DN-DBC1, which lacks the N-terminal region. We show that DN-DBC1 appears in quiescent cells and is produced by proteolytic cleavage of pre-existing DBC1. Moreover, the balance between these two forms is dynamically regulated *in vitro and in vivo*. We mapped the missing sequence in DN-DBC1, showing that this protein lacks the first 124–138 amino acidic residues, which includes the S1-like domain. Importantly, we show that DN-DBC1 does not bind to SIRT1. Finally, we show that DBC1 and DN-DBC1 are differentially regulated *in vivo* during liver regeneration and that DBC1 KO mice have delayed liver regeneration after partial hepatectomy by a mechanism probably involving dysregulation of normal cell cycle progression.

Until now, most of the evidence regarding the role of DBC1 in the control of cell cycle were obtained *in vitro* using transformed cells. Understanding how DBC1 is regulated in normal cells and tissues is vital not only to assess its function in normal physiological conditions but also in cancer. Our results show for the first time that DBC1 is involved in normal cell cycle control *in vitro* and *vivo*. We found that, in quiescent cells *in vitro* as well as in resting liver cells, there is a short form of DBC1 (DN-DBC1), which is rapidly down-regulated when quiescent cells re-enter into the cell cycle. Proteolytic processing of DBC1 under different conditions has been previously proposed. Two previous reports suggested the appearance of a short form of DBC1 which, under their experimental conditions was linked to apoptosis^32,33^. Different to their findings, we found that at least in quiescent cells, induction of apoptosis by etoposide leads to the loss of DN-DBC1 rather than its appearance. We cannot exclude that DN-DBC1 or other different proteolytic processing of the protein could be produced in other contexts of cellular apoptosis. However, we believe that this novel form of DBC1 is mainly expressed in quiescent cells and that this regulation may be lost in transformed malignant cells. Importantly, DN-DBC1 is also lost in a cellular model of cellular senescence. Although further work should be conducted using other models of cellular senescence, our findings suggest that the DBC1/DN-DBC1 ratio could be a marker of different types of cell cycle arrest. In fact, it has been recently shown that SIRT1 regulates SASP^37^ suggesting that down-regulation of DN-DBC1 may be required for the establishment of the SASP in senescent cells. The fact that DN-DBC1 is lost both in quiescent cells that undergo apoptosis and in senescent cells suggest that DN-DBC1 is not only a marker of quiescent cells. We speculate that down-regulation of DN-DBC1 is a key regulatory event in the decision of the cellular fate upon leaving quiescence, either to re-enter cell cycle, to undergo apoptosis, or to go into cellular senescence.

DN-DBC1 appears to be an inactive form of the protein, at least in terms of SIRT1 inhibition. It is puzzling the fact that in some cases, the presence of DN-DBC1 correlates with clear decay in DBC1, while in other instances this correlation is not evident. Although we cannot provide an answer for this apparent inconsistency, we believe that it could be due to different equilibrium dynamics among cell types or perhaps a reflection of cell culture heterogeneity. This issue needs further investigation and is important in order to understand the dynamics of DN-DBC1 and its biological functions. Mapping of the cleavage sequence of DBC1 showed that DN-DBC1 is missing the first 124–138 residues, thus losing only the S1-like domain. Still, DN-DBC1 was not able to bind SIRT1, which is in line with a recent report showing that DBC1 uses the S1-like binding domain in order to bind and regulate SIRT1^15^. The fact that DN-DBC1 does not bind SIRT1 has important implications in terms of understanding how the SIRT1-DBC1 interaction is regulated. It was originally reported that DBC1 binds SIRT1 via its LZ domain^2,3^. It was later suggested that the N-terminal sequence of DBC1 is also important for SIRT1 binding, but no definitive evidence was provided^13^. Our findings show that a naturally existing form of DBC1 that does not bind SIRT1 is regulated during the cell cycle and it is mainly present during G0, implicating that SIRT1 might not be targeted by DBC1 in quiescent cells.

It is worth noticing that most of the binding partners and molecular targets of DBC1, like ER, p53, BRCA1 have been mapped to bind DBC1 through its LZ and N-terminal domains^1^. Based on this data, it is plausible that DN-DBC1 might not only lose its ability to bind SIRT1 but also other targets. In fact, BRCA1 and p53 are key regulators of cell cycle control with major roles in cancer^38,39^. Our experimental evidence cannot rule out a putative role of either BRCA1 or p53 in the DBC1-dependent effects observed when cells leave quiescence. Interestingly, besides being targets of DBC1, both p53 and BRCA1 activity are directly regulated by SIRT1 activity^40,41^. The interplay among all these proteins is complex and understanding its regulation goes beyond the aim of this work. However, the possibility that DN-DBC1 is also inactive in regulating p53 and BRCA1 may have major implications in cell cycle control and cancer, so it will be further investigated. In addition, the S1-like binding domain of DBC1 seems to go beyond the regulation of protein-protein interaction. It was shown recently that DBC1 binds to the lncRNA MALAT1 regulating its biological activity^14^. The binding of DBC1 to MALAT1 was mapped to the N-terminal domain of DBC1. It is possible that the specific loss of the S1-like RNA binding domain in DBC1 has important implications in the regulation of MALAT-1 and possibly other RNAs.

We show here that DBC1 KO mice have impaired liver regeneration. In fact, we show that SIRT1 activity inhibition observed in WT mice during early steps of liver regeneration is not present in livers form DBC1 KO mice. Interesting, a recent report using transgenic SIRT1 overexpressing mice showed impaired liver regeneration compared to WT mice^27^. Our results are in accordance with those findings and although we do not provide a direct link between SIRT1 over-activation and altered liver regeneration in DBC1 KO mice, it is plausible that this could be the case. In addition, since DBC1 has many other protein targets besides SIRT1, we cannot rule out that other mechanisms exceeding SIRT1 regulation are taking place in the DBC1 KO mice. It is interesting the fact that livers from DBC1 KO mice show a delay in cell cycle progression after S phase, with a decreased percent of mitotic cells compared to WT mice, suggesting that liver cells get delayed in the G2/M transition. Similar results were obtained in MEFs. Importantly, previous reports using U2OS cells have shown Dbc1 to be necessary for a correct G2/M transition through a yet unknown mechanism^42^. It is possible then, that during liver regeneration, DBC1 KO cells are delayed in the G2/M transition of the cell cycle, slowing down cell cycle and affecting mass liver recovery. Further investigation will be needed in order to clearly determine if this is the mechanism operating.

Finally, our results also bring new insights into the role of DBC1 in the regulation of cell cycle, which are likely to be relevant to understand the reported function of DBC1 in cancer. Traditionally it has been proposed that absence of DBC1 promotes tumorigenesis^6,7,42–45^, although this view may not be accurate for all cancers^46^. In fact, many studies in cancer have been conducted using antibodies against the N-terminal sequences of DBC1^45–53^, raising the possibility that DBC1 expression could have been underestimated because of failure to detect DN-DBC1. Thus, we believe that the role of DBC1 in cancer and its value as a predictor marker may need to be re-evaluated.

## Conclusions

This work contributes substantial novel findings about the role of DBC1 in the regulation of liver regeneration, providing novel insights about the role of DBC1 in the regulation of liver function. Our findings also bring knowledge about the role of DBC1 in cell cycle regulation, which we believe is important to understand its function not only in liver regeneration but also in cancer. Additionally, the fact that DN-DBC1 no longer binds to SIRT1 brings new questions about the regulation of the SIRT1/DBC1 interaction and possibly about other molecular targets of DBC1. Finally, whether DN-DBC1 is just an inactive form of DBC1 or it has acquired novel roles different from those of DBC1 will need to be further investigated.

## Supplementary information

Supplementary information

Raw data
