# Supplementary material for: A novel form of Deleted in breast cancer 1 (DBC1) lacking the N-terminal domain does not bind SIRT1 and is dynamically regulated in vivo
Source: Sci Rep. 2019 Oct 7;9:14381. doi: 10.1038/s41598-019-50789-7 (PMC6779753; doi:10.1038/s41598-019-50789-7)
Supplement: Supplementary file 1 — Supplementary information [file 41598_2019_50789_MOESM1_ESM.pdf]

A novel form of Deleted in breast cancer 1 (DBC1) lacking the N-terminal domain does not bind SIRT1 and is dynamically regulated *in vivo*.

Leonardo Santos[1], Laura Colman[1], Paola Contreras[1,3], Claudia C.S. Chini[2], Adriana Carlomagno[1], Alejandro Leyva[4], Mariana Bresque[1], Inés Marmisolle[9], Celia Quijano[9], Rosario Durán[4], Florencia Irigoín[5,6], Victoria Prieto-Echagüe[5], Mikkel H. Vendelbo[9], José R. Sotelo-Silveira[8], Eduardo N. Chini[2], Jose L. Badano[5], Aldo J. Calliari[1,7], Carlos Escande[1*]

Supplementary information

Supplementary Figure S1

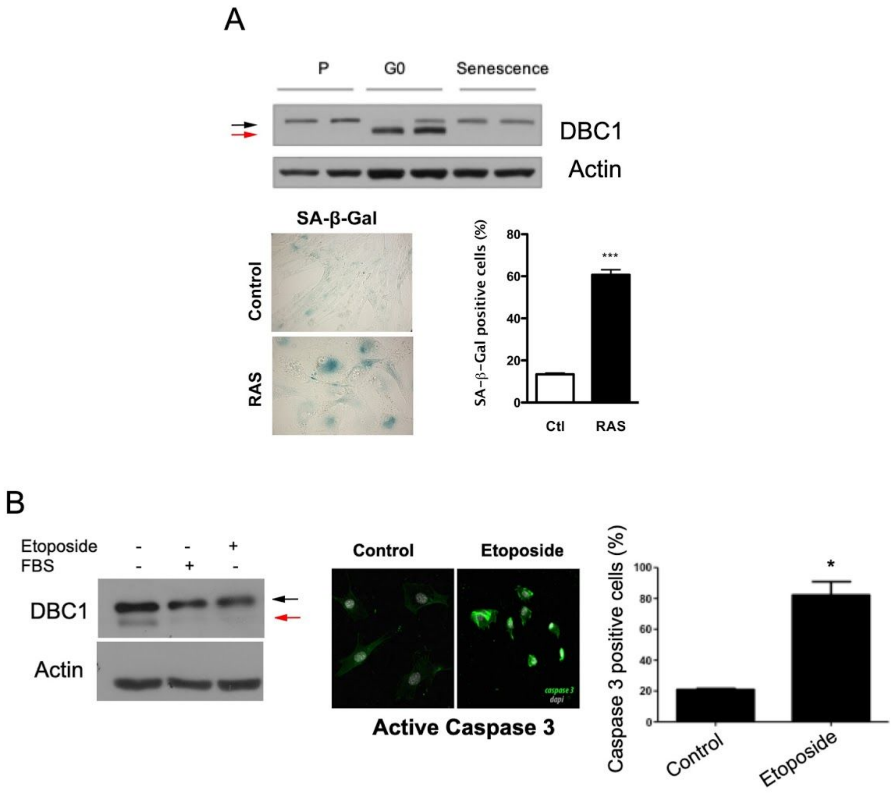

A) IMR90 cells were maintained in a sub-confluent proliferating state (P), quiescent by 10 days of confluence (G0), or Senescent by overexpression of the oncogene RAS (Senescence). Senescence Associated (SA) ß-galactosidase activity assay shows the induction of cellular senescence by RAS. P value<0.0001 two-tailed unpaired t-test (n=3) **B)** WT MEFS were incubated with 0.1% FBS, 10% FBS, or with 0.1% FBS + 150 uM Etoposide for 1 hour to induce apoptosis. DBC1 cleavage is absent in etoposide-treated cells**.** Center, representative image for caspase 3 staining in untreated and treated cells. Right, quantitation of Caspase 3 activation during etoposide treatment. P value<0.05 two-tailed unpaired t-test (n=3)

Supplementary Figure S2

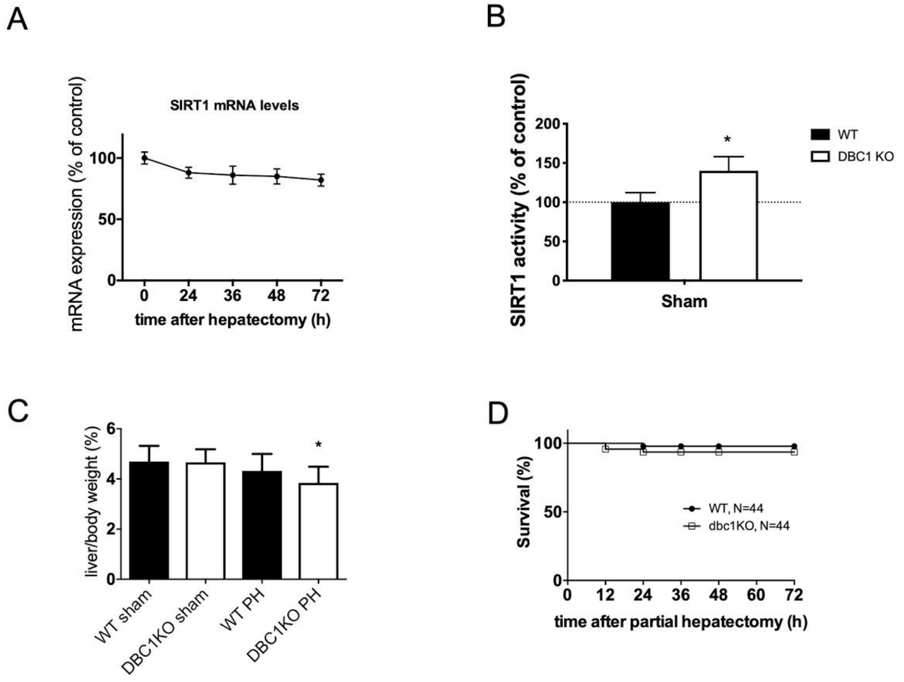

Supplementary Figure S2. A) Time course of SIRT1 mRNA expression in the liver after partial hepatectomy. **B)** SIRT1 activity in the liver from WT and DBC1 KO mice in basal (sham mice) conditions. SIRT1 activity was measured from isolated nuclei from WT and DBC1 KO. * $p<0.05$, t-test. **C)** Liver mass recovery WT and DBC1 KO 7 days after partial hepatectomy and compared with control (sham) mice. *$p<0.05$ compared to WT Sham and DBC1 KO Sham. One-way ANOVA. **D)** Kaplan-Meier survival curves of WT and DBC1 KO mice after partial hepatectomy surgery.

Supplementary Figure S3

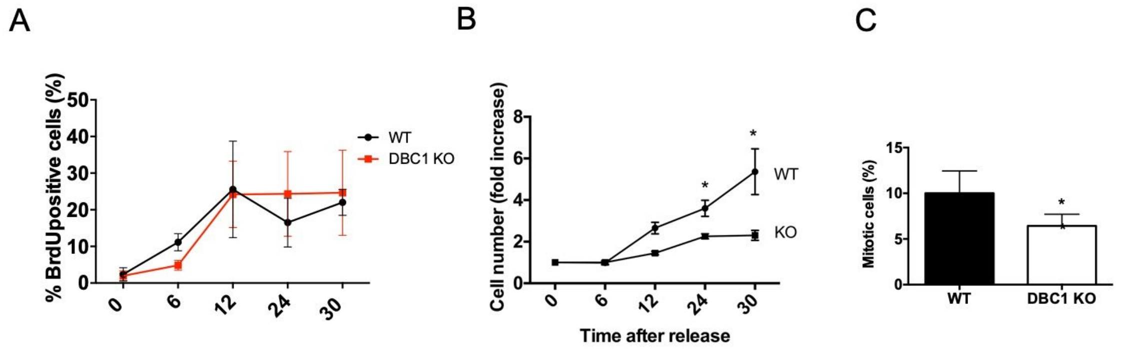

Supplementary Figure S3. Primary DBC1 KO MEFs show altered cell cycle progression after leaving quiescence. Primary MEFs (passages 2-4) were synchronized by incubation in 0.1% FBS for 48 hours. After that time, cell media was replaced to 10% FBS and cells were recovered at the indicated time points. **A)** Cells were given a 2 hour BrdU (10μM) pulse before the indicated time points. **B)** Quantitation of total cell number after serum deprivation release. Total cell number was counted and normalized to cell count at time 0. * mean $p<0.05$ and $p<0.01$ respectively, two-tailed unpaired t-test for each time point (n=4). **C)** Mitotic index (calculated as the fraction of cells with chromosomal condensation by DAPI staining) in WT and DBC1 KO MEFs 6 hours after addition of FBS. * $p<0.05$, two-tailed unpaired t-test (n=4).

Supplementary Methods

Splicing pattern of DBC1 in mice and human.

Assessing splicing in mice and humans transcripts was performed on deep RNA-seq data from 90 million reads from normal liver transcriptomes (30) and from the CHESS database respectively (https://www.biorxiv.org/content/early/2018/05/29/332825, CHESS 2.1 is a comprehensive set of human genes based on nearly 10,000 RNA sequencing experiments produced by the GTEx project). In both cases de novo assembly of transcripts and reconstruction of splicing forms was carried out using Hisat/Stringtie method (Pertea, M., Kim, D., Pertea, G. M., Leek, J. T. & Salzberg, S. L. Transcript-level expression analysis of RNA-seq experiments with HISAT, StringTie and Ballgown. *Nat Protoc* **11**, 1650-1667. doi:10.1038/nprot.2016.095). In the case of CHESS 2.1 data, we studied the splice isoforms assembled and available http://ccb.jhu.edu/chess/. In silico translation of these splice variants did not produce proteins of the size detected here. Visualization of splice variants were rendered using IGV (Robinson, J. T. *et al.* Integrative genomics viewer. *Nat Biotechnol* **29**, 24-26, doi:10.1038/nbt.1754) or CLC Genomics Worbench (ver 11, Qiagen).
