# Supplementary material for: A novel form of Deleted in breast cancer 1 (DBC1) lacking the N-terminal domain does not bind SIRT1 and is dynamically regulated in vivo
Source: Sci Rep. 2019 Oct 7;9:14381. doi: 10.1038/s41598-019-50789-7 (PMC6779753; doi:10.1038/s41598-019-50789-7)

A novel form of Deleted in breast cancer 1 (DBC1) lacking the N-terminal domain does not bind SIRT1 and is dynamically regulated *in vivo*.

Leonardo Santos[1], Laura Colman[1], Paola Contreras[1,3], Claudia C.S. Chini[2], Adriana Carlomagno[1], Alejandro Leyva[4], Mariana Bresque[1], Inés Marmisolle[9], Celia Quijano[9], Rosario Durán[4], Florencia Irigoín[5,6], Victoria Prieto-Echagüe[5], Mikkel H. Vendelbo[9], José R. Sotelo-Silveira[8], Eduardo N. Chini[2], Jose L. Badano[5], Aldo J. Calliari[1,7], Carlos Escande[1*]

Supplementary information – Raw data

WB Figure 1

Figure 1A

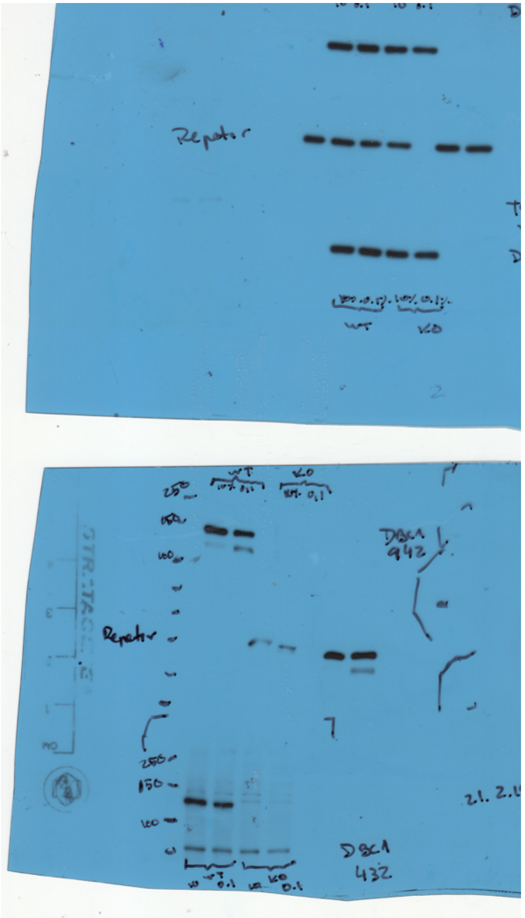

Figure 1B

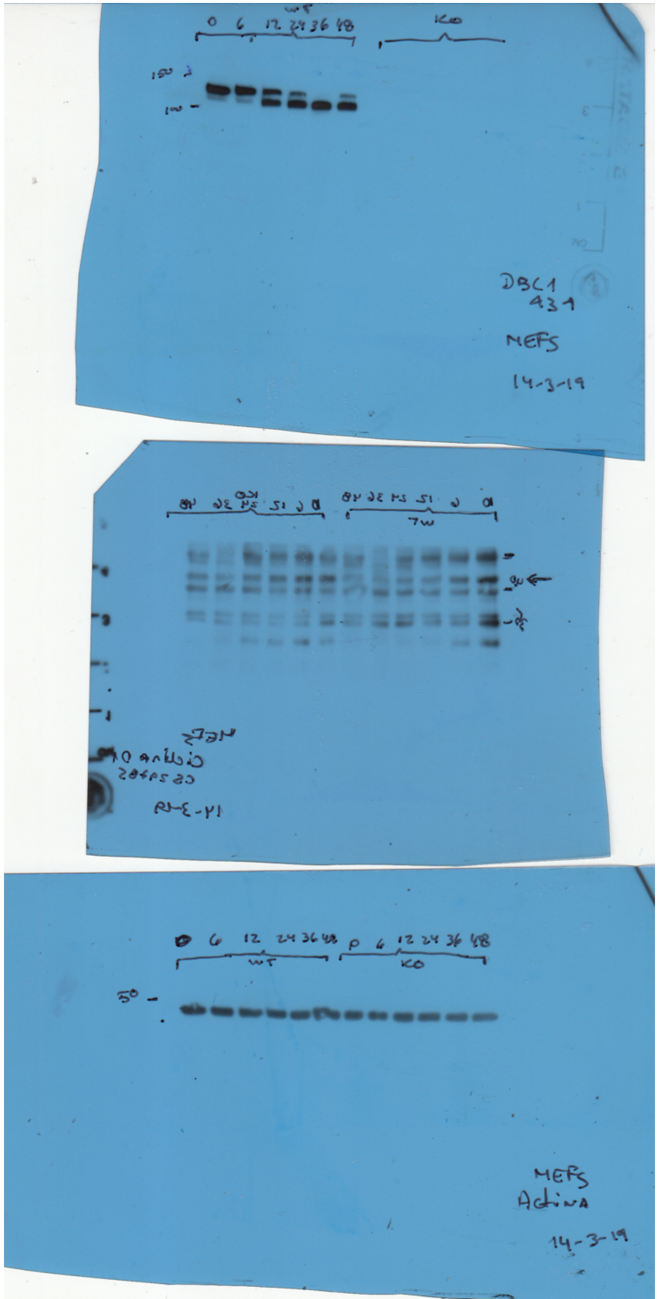

WB Figure 1

Figure 1C

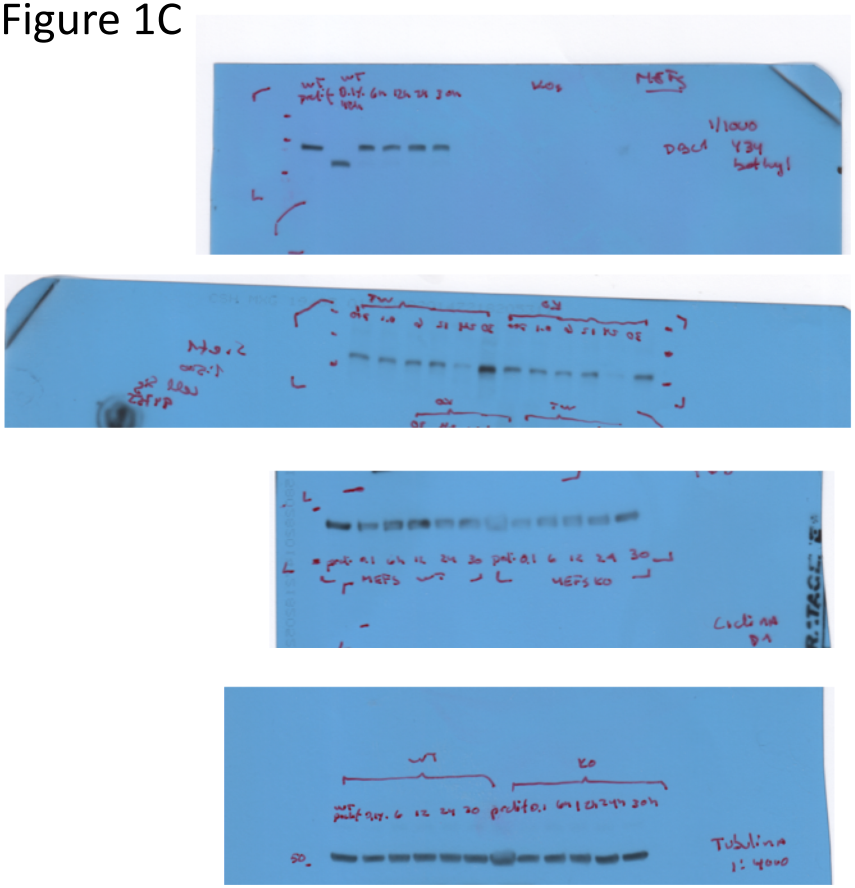

WB Figure 2

Figure 2A

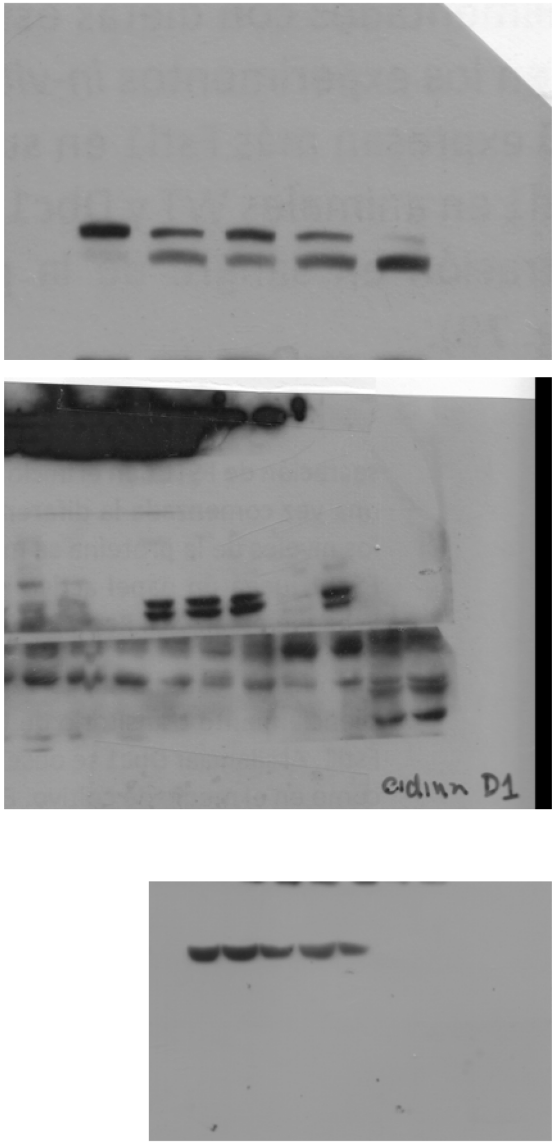

Figure 2B

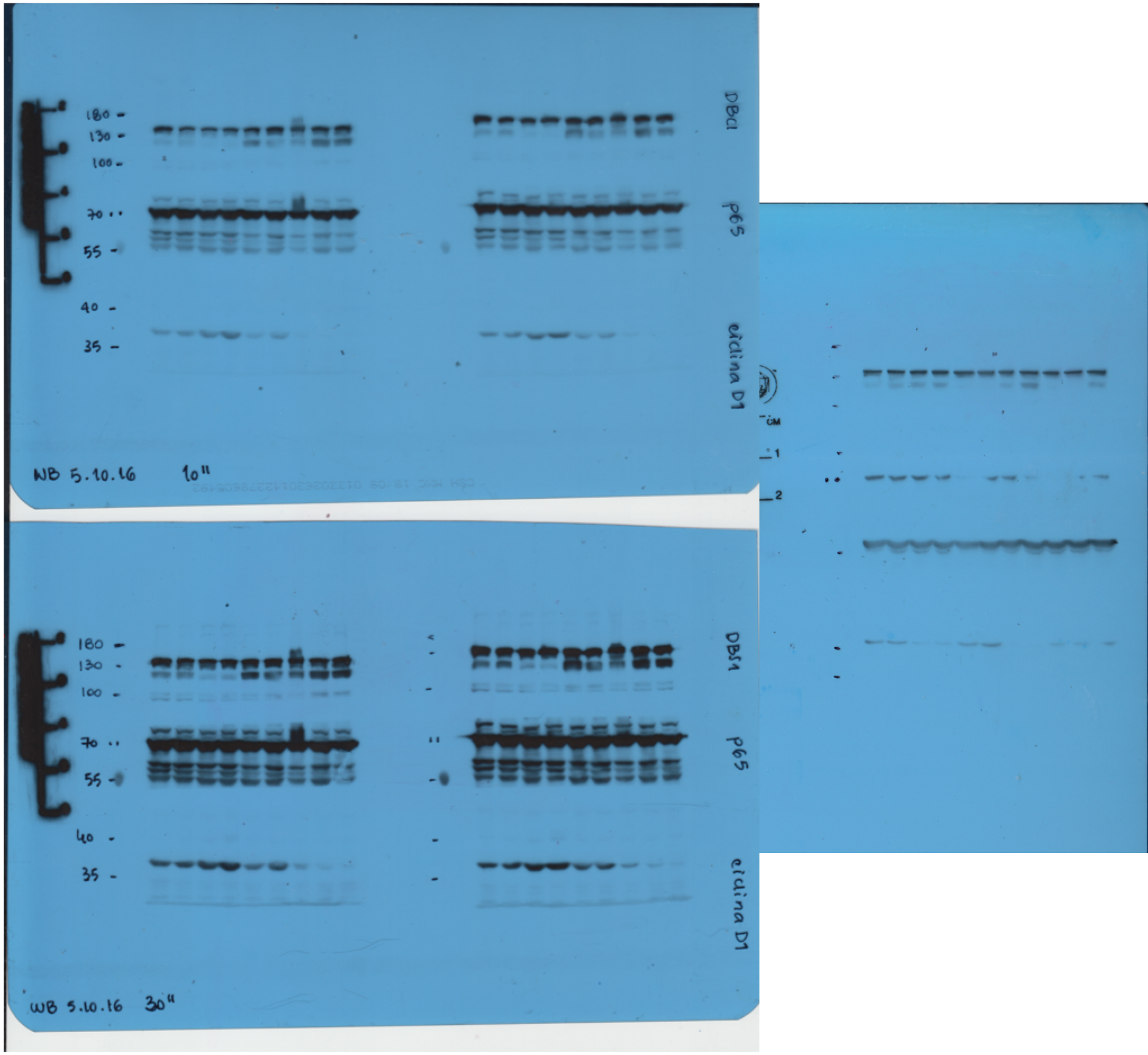

WB Figure 2

Figure 2C

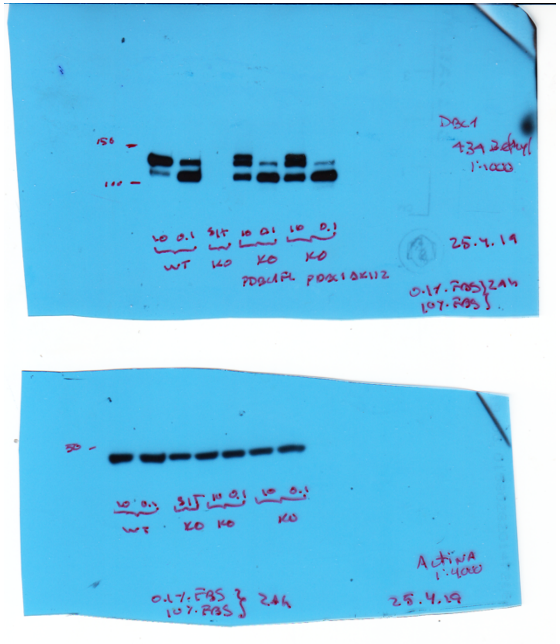

Figure 2F

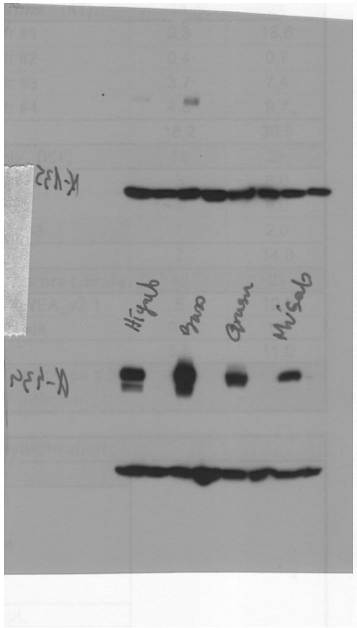

Figure 2G

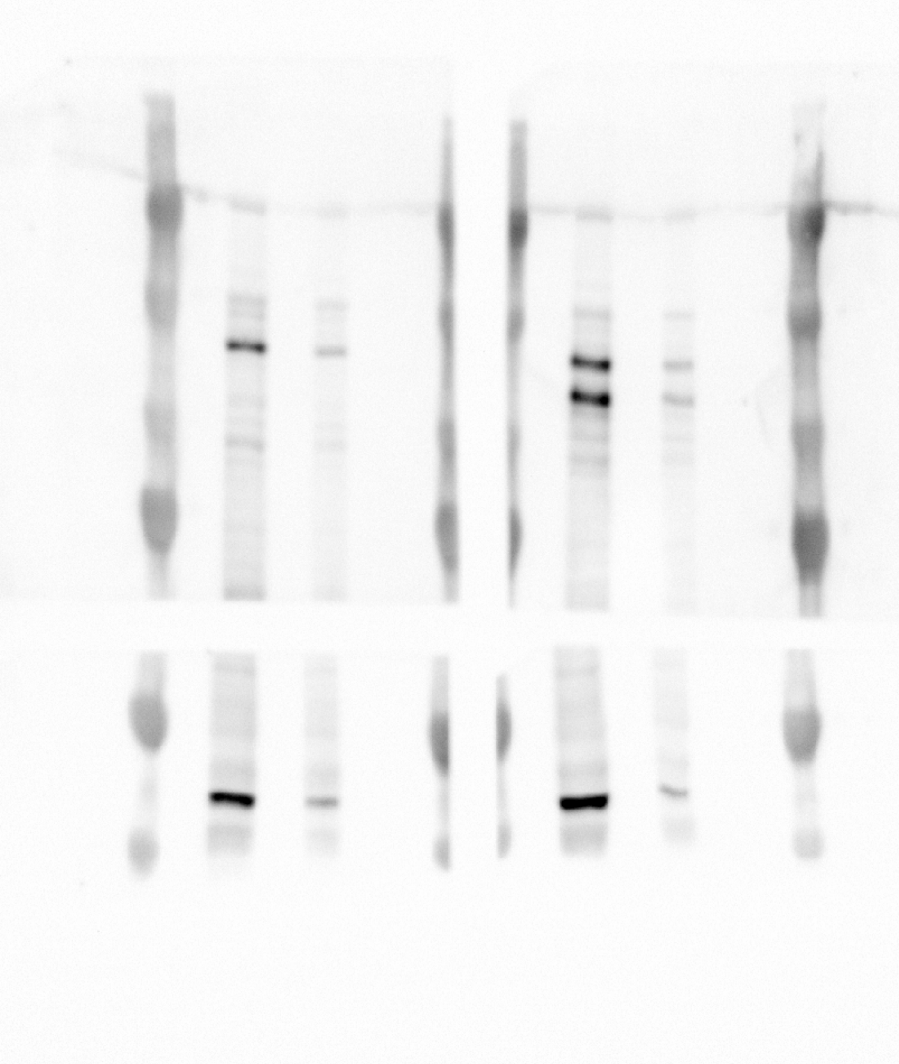

WB Figure 3

Figure 3D

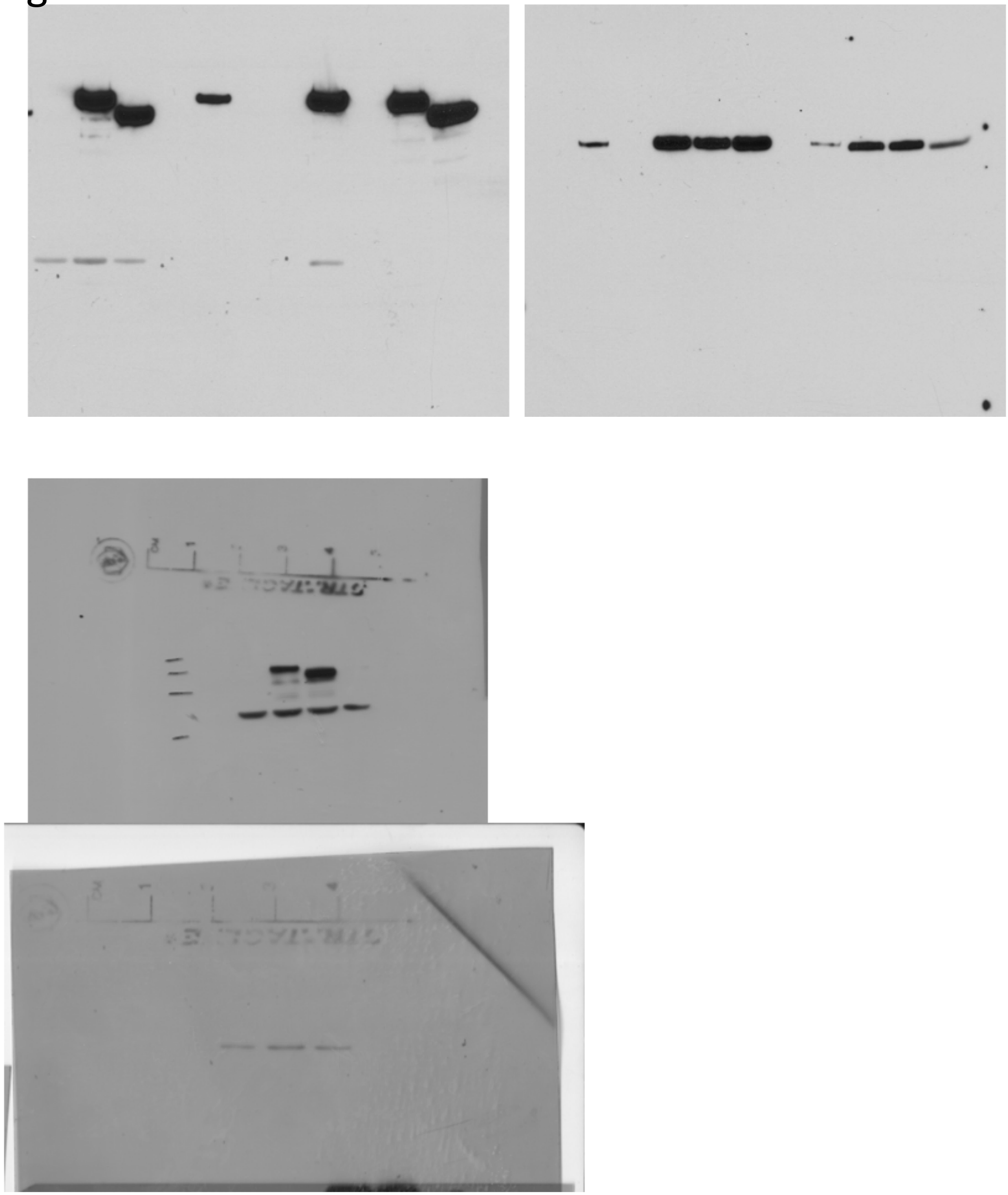

WB Figure 3

Figure 3F

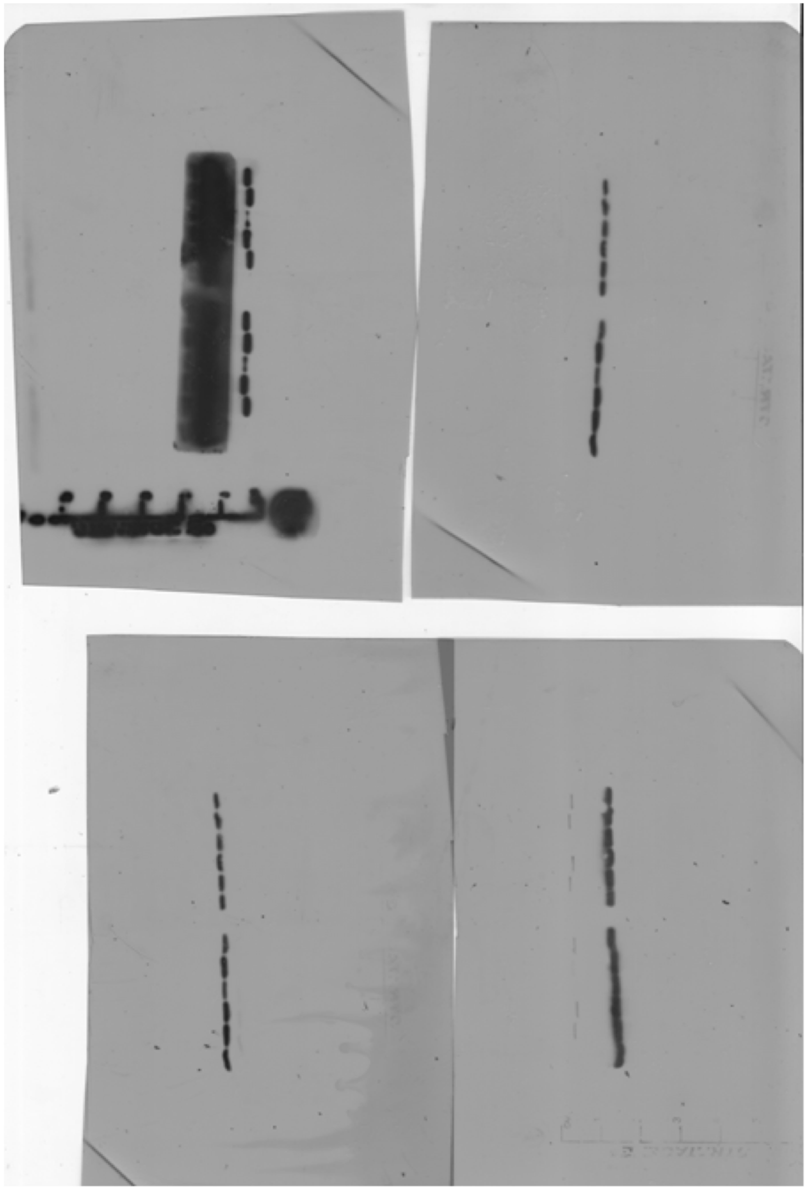

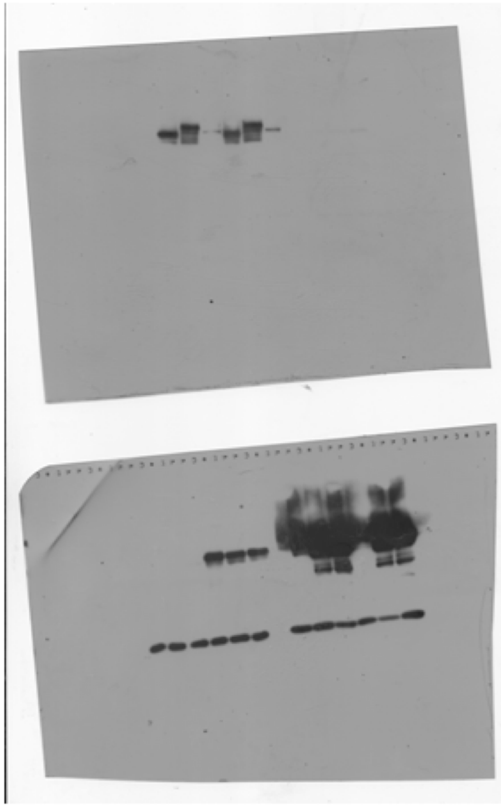

WB Figure 3

Figure 3E

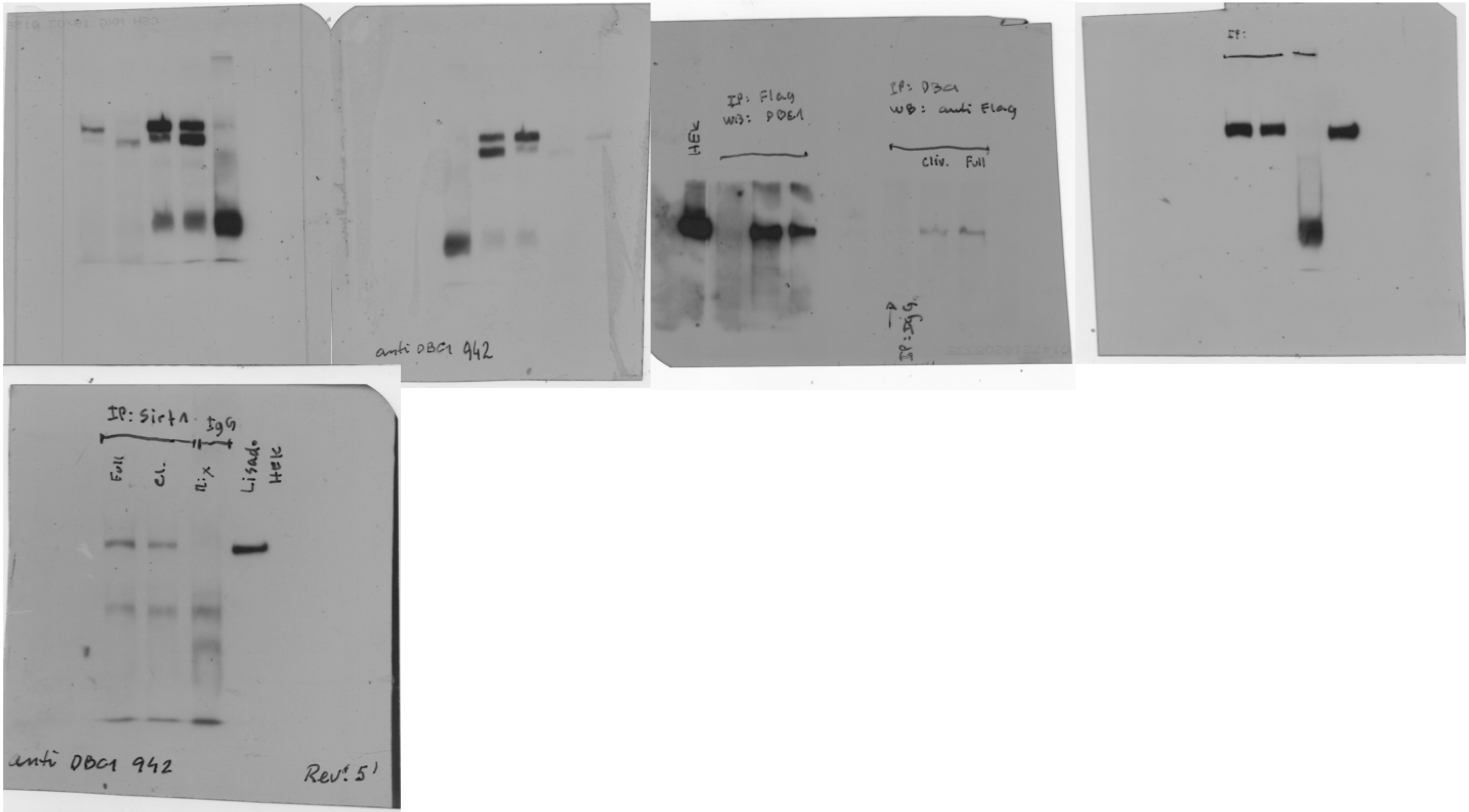

WB Figure 4

Figures 4A-B

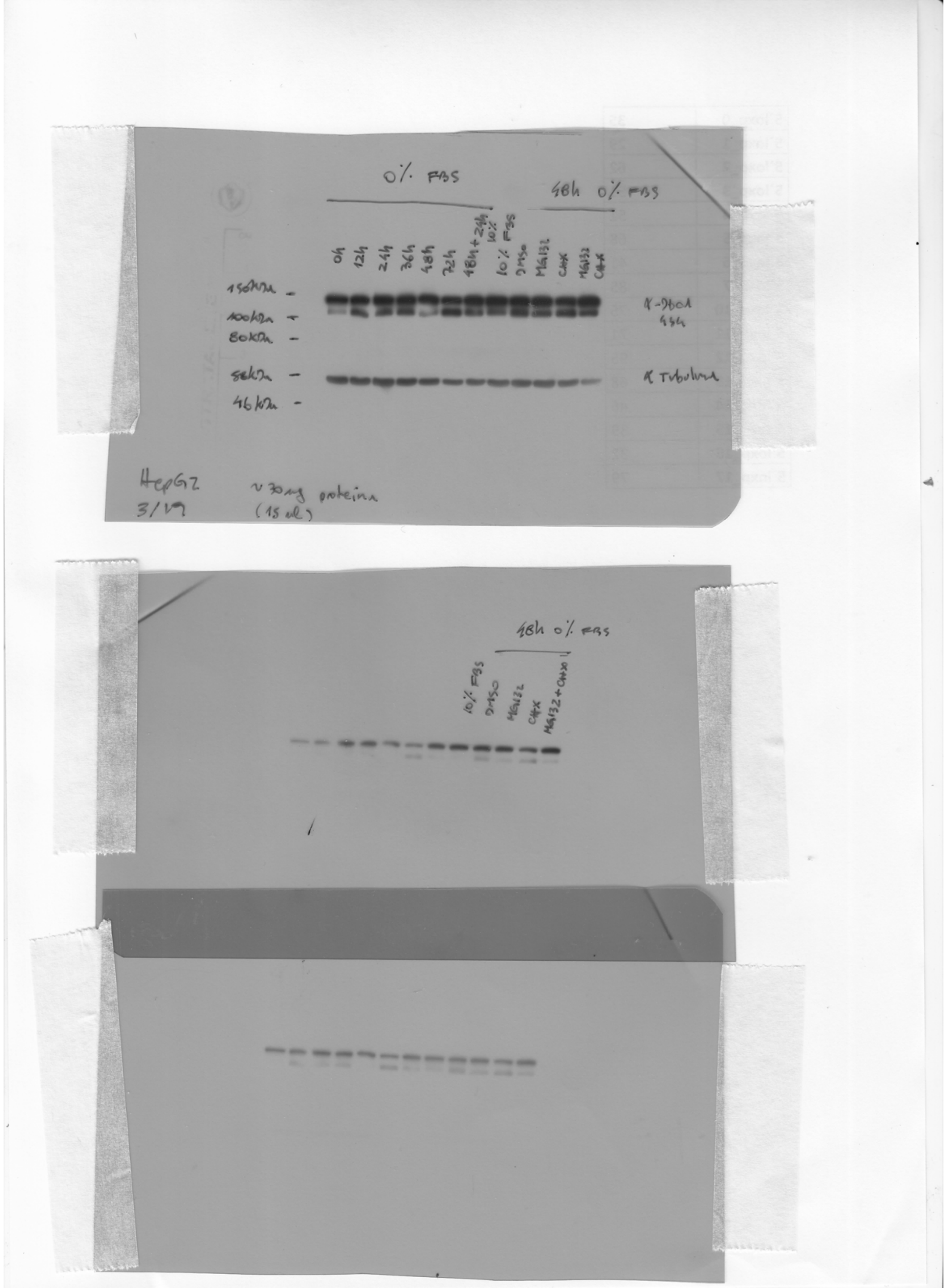

WB Figure 4

Figures 4C-D

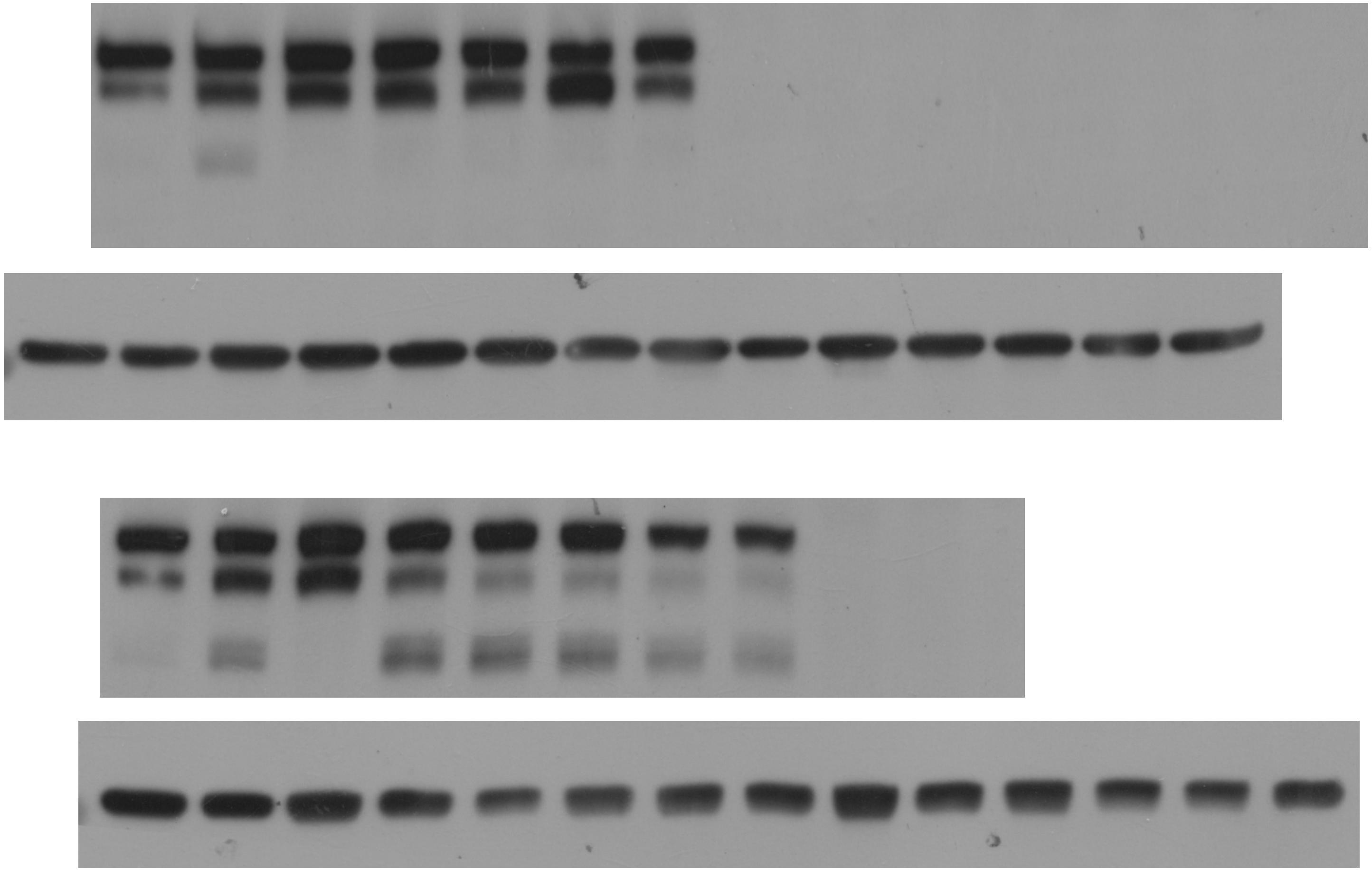

WB Figure 4

Figure 4F

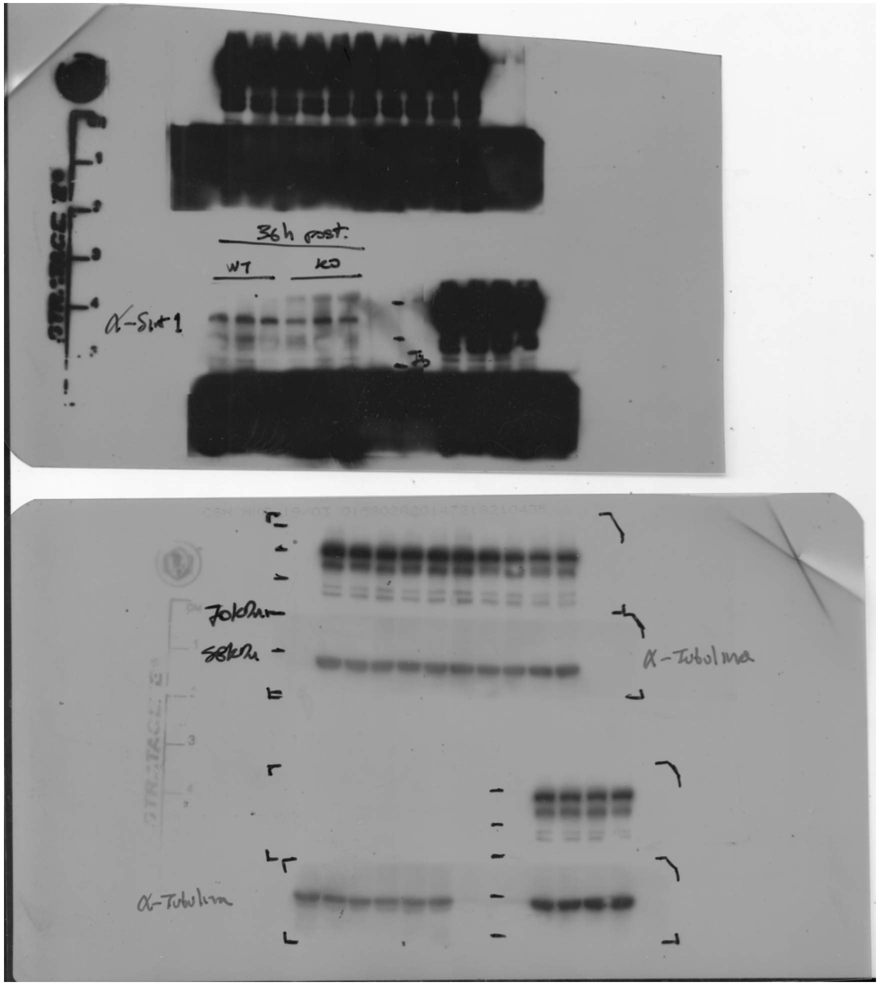

Figure 4G

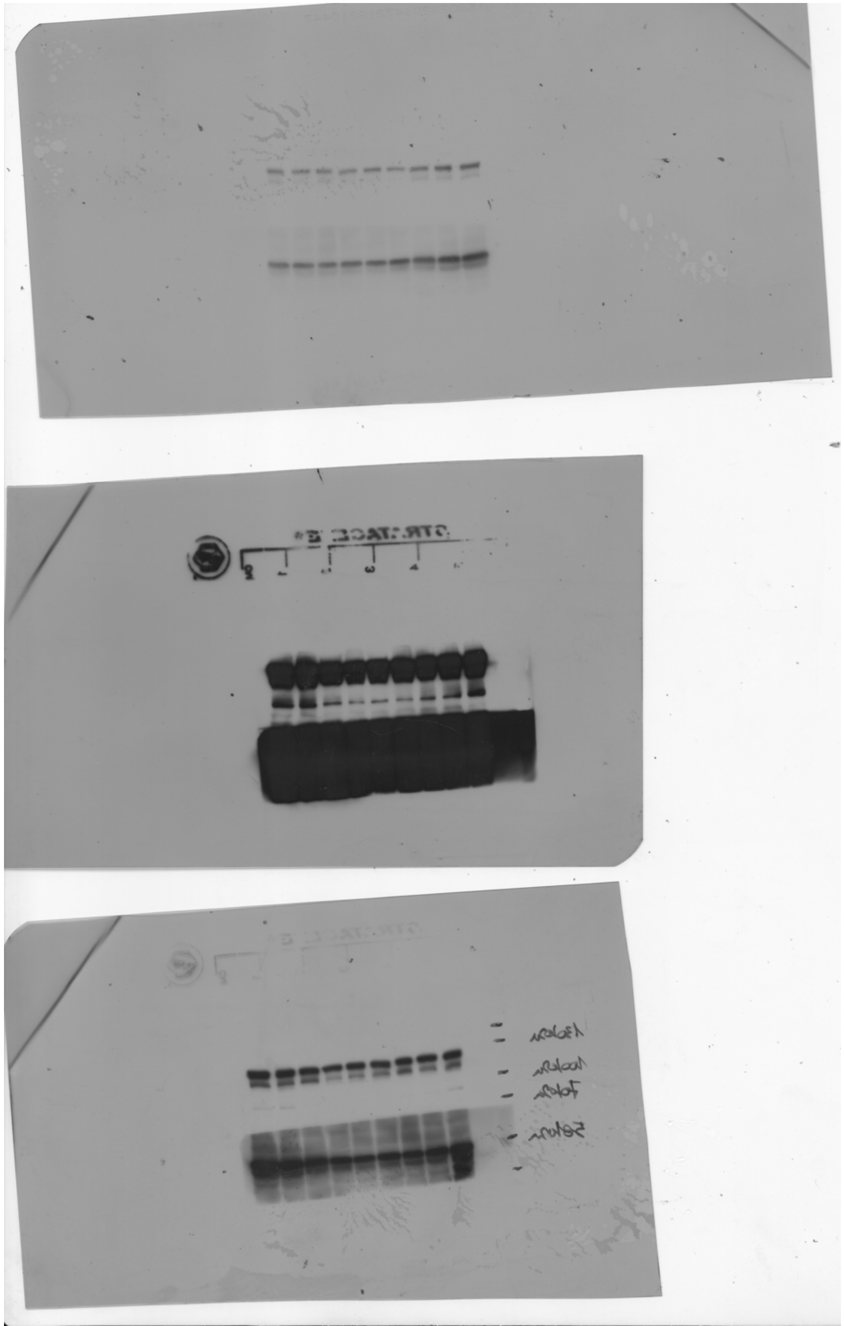

Supplementary Figure 1

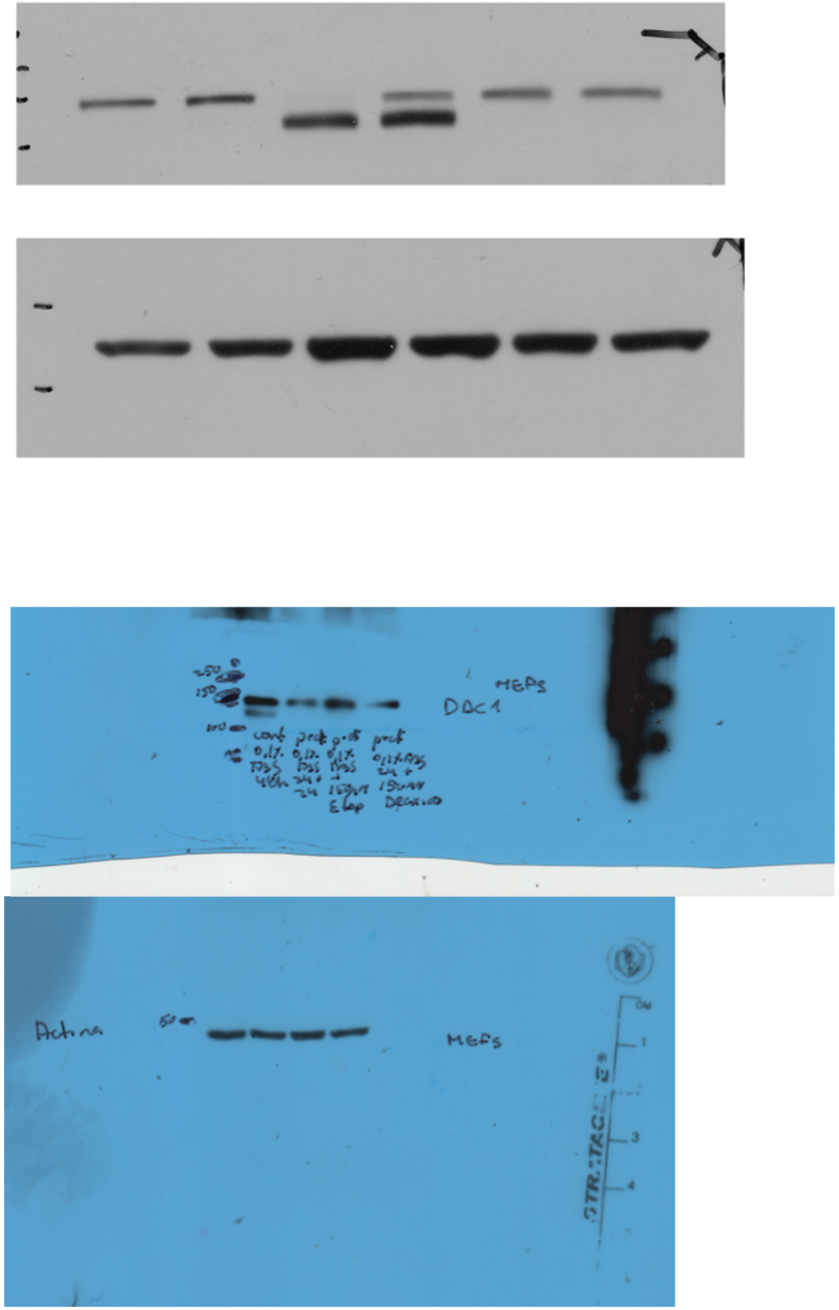

Supplement: Supplementary file 2 — Raw data [file 41598_2019_50789_MOESM2_ESM.pdf]
